# Protracted metallogenic and magmatic evolution of the Kirazlı epithermal Au-Ag and porphyry Cu deposits, Biga Peninsula, NW Turkey: evidence from zircon U-Pb, muscovite ^40^Ar/^39^Ar, and molybdenite Re-Os geochronology

**DOI:** 10.1007/s00126-023-01235-2

**Published:** 2023-12-18

**Authors:** Ali Aluç, İlkay Kuşcu, Alexey Ulyanov, David Selby, Clémentine Antoine, Richard Spikings, Robert Moritz

**Affiliations:** 1 Department of Geological Engineering, Mugla Sıtkı Koçman University, 48000 Muğla, Türkiye; 2 Department of Earth Sciences, Mineral Resources and Geofluids, University of Geneva, 1206 Geneva, Switzerland; 3 Ortaköy mah. Diğer Küme Evleri, No. 349/7, 48000 Muğla, Türkiye; 4 Faculty of Geosciences and Environment, Géopolis, University of Lausanne, 1022 Lausanne, Switzerland; 5 Durham University, Department of Earth Sciences, DH1 3LE Durham, United Kingdom

**Keywords:** High-sulfidation epithermal, Porphyry Cu, U-Pb – ^40^Ar/^39^Ar – Re-Os geochronology, Kirazlı, Biga Peninsula

## Abstract

The Kirazlı deposit is located at the center of the Biga Peninsula metallogenic province, in a geological setting characterized by an extensional tectonic environment. A NNW-SSE trending high-sulfidation (HS) orebody with a total reserve of 33.86 Mt @ 0.69 g/t Au and 9.42 g/t Ag lies beneath the Kirazlı Main zone. A porphyry Cu orebody hosted by Eocene intrusive and volcanic rocks has been intersected by drilling within its vicinity. The HS epithermal deposit is hosted by a partly silicified and brecciated Oligocene volcanic and volcaniclastic sequence consisting mainly of basaltic andesite lava flow and lithic/crystal tuff. Lithogeochemistry and zircon U-Pb radiometric ages allow us to distinguish three distinct high-K calc-alkaline magmatic events at *ca.* 41, 38, and 32 Ma, sourced by metasomatized mantle melts, which have interacted with the crust during their ascent. Porphyry Cu mineralization took place at 36.7 ± 0.4 Ma (muscovite ^40^Ar/^39^Ar age) with subsequent re-opening and base metal deposition. Crosscutting quartz-pyrite-molybdenite veins were emplaced at 33.6 ± 0.2 Ma (molybdenite Re-Os age), and followed by the HS epithermal Au-Ag event at *ca.* 31 Ma, based on a previous study. Our radiometric data indicate that the Kirazlı deposit has recorded a long-lasting Cenozoic magmatic and metallogenic evolution during about 10 Myr. Our study demonstrates that successive, independent, and overprinting, but genetically unrelated, HS epithermal precious metal, hydrothermal Mo, base metal, and porphyry Cu systems have been active at the same location during protracted extensional tectonics of the Biga Peninsula.

**Supplementary Information:**

The online version contains supplementary material available at 10.1007/s00126-023-01235-2.

## Introduction

**Fig. 1:**
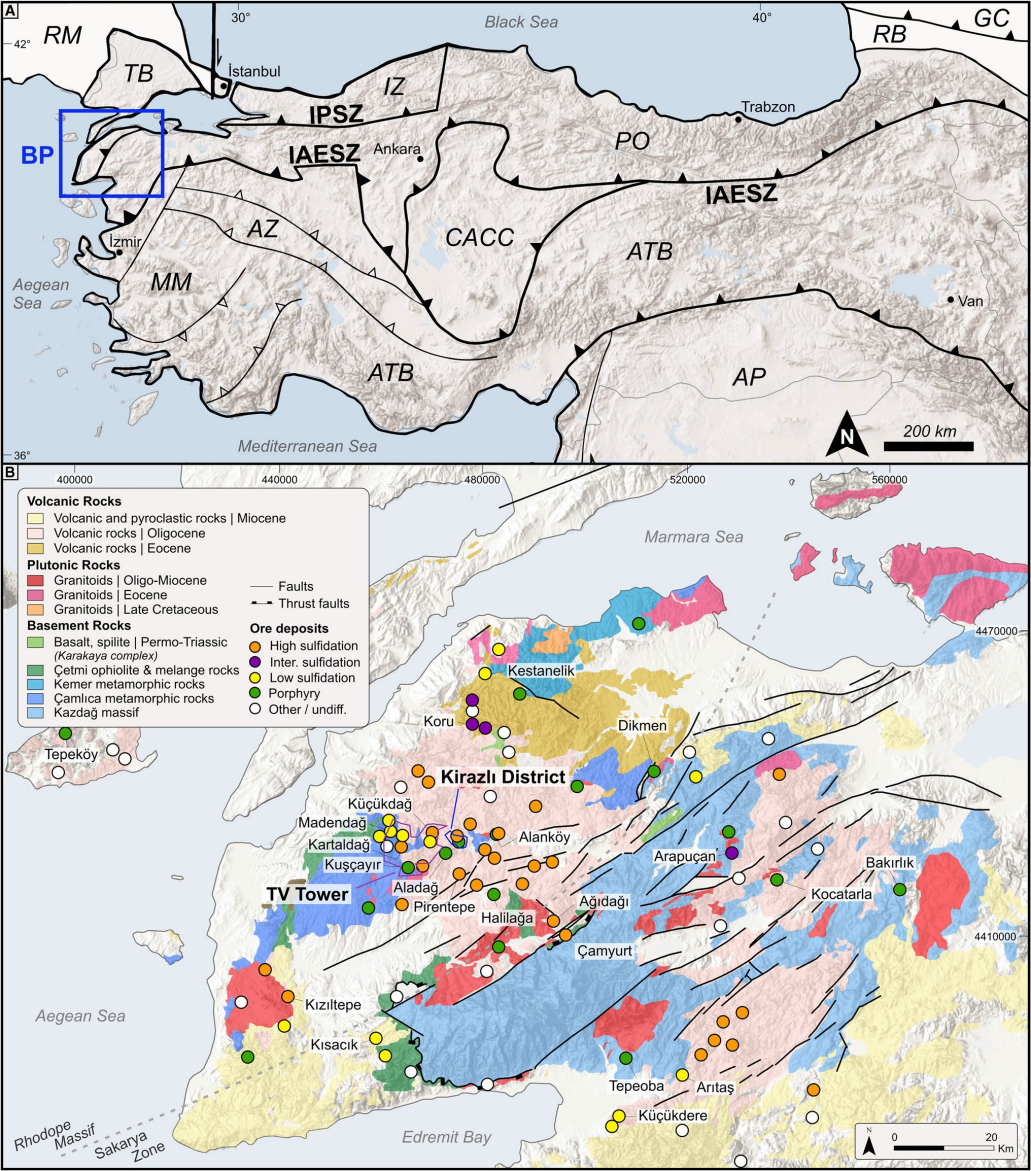
(**A**) Simplified map showing the tectonic units of Turkey and surrounding regions (modified from Okay and Tuysuz 1999; AZ, Afyon Zone; ATB, Anatolide-Tauride Block; AP, Arabian Platform; MM, Menderes Massif; CACC, Central Anatolian Crystalline Complex; IAESZ, Izmir-Ankara-Erzincan Sture Zone; PO, Pontides; IPSZ, Intra-Pontide Sture Zone; IZ, Istanbul Zone; TB, Thrace Basin; RM, Rhodope Massif; RB, Rioni Basin; GC, Greater Caucasus). (**B**) Geological map showing volcanic, plutonic, basement rocks, main structural units, and the major ore deposits and occurrences of the Biga Peninsula (simplified from 1:25 K Geological map of Turkey (MTA), the coordinates for (A) in decimal degrees and for (B) in UTM WGS 1984 Zone 35N)

The Biga Peninsula is a metallogenic gold-copper province in the northwestern part of Turkey (Fig. 1A), which hosts numerous epithermal Au-Ag±Cu and porphyry Au-Cu-Mo deposits and prospects (Fig. 1B, ESM 1 Table S1), where epithermal systems predominate, in particular, high-sulfidation (HS) systems (Yiğit 2012; Fig. 1B) These deposits and prospects are associated with arc magmatism, accompanied and overprinted by extensional and strike-slip tectonics from the early Eocene to the middle Miocene (*ca.* 52–18 Ma, Yiğit 2012; Kuşcu et al. 2019a). Sánchez et al. (2016) suggested that the porphyry Cu systems and many epithermal deposits formed synchronously with regional-scale post-orogenic extension at the Biga Peninsula.

In some metallogenic provinces, it has been documented that high-sulfidation (HS) epithermal systems were contemporaneous with porphyry Cu deposits (PCDs), with both having formed within a single magmatic-hydrothermal system and centered on a single magmatic intrusion (Arribas Jr et al. 1995; Hedenquist et al. 1998; Sillitoe 2010). By contrast, other studies (Setterfield et al. 1992; Losada-Calderòn et al. 1994; Losada-Calderòn and McPhail 1998; Marsh et al. 1997; Rohrlach 2003; Masterman et al. 2005) have yielded significant age differences between PCDs and adjacent HS epithermal systems, therefore demonstrating overprinting hydrothermal events related to physically and temporally distinct magmatic systems.

In the Biga Peninsula, several spatially associated PCDs and HS epithermal systems (e.g., Aladağ, Halilağa) with unclear temporal relationships have been reported (Yiğit 2012; Hetman et al. 2014; Smith et al. 2014; Brunetti 2016; Kuşcu et al. 2019b. Recent detailed studies on the geology and the temporal association of overprinting mineralization styles in individual deposits have questioned any coeval character of PCDs and HS epithermal systems in the Biga Peninsula, and have opened the debate about spatially associated, but genetically unrelated PCDs and HS epithermal systems (Brunetti 2016; Leroux 2016; Aluç et al. 2021). The main reasons for this debate are (1) poorly understood overprinting multiphase hydrothermal processes, (2) a lack of reliable absolute ages of the host rocks, mineralization, and alteration, and (3) a lack of detailed information about the volcanic stratigraphy and correlations, due to regional hydrothermal alteration blurring the original nature of protoliths, as well as post-depositional extensional tectonic events.

The Kirazlı deposit at the center of the Biga Peninsula (Fig. 1B) hosts an HS epithermal Au-Ag orebody dated at 30.7 ± 1.5 Ma (alunite-rich whole-rock ^40^Ar/^39^Ar age, Yiğit 2012), which is adjacent to a porphyry Cu orebody, found by a drilling exploration program (Cormier et al. 2017). The hydrothermal systems are hosted by Eocene and Oligocene magmatic rocks emplaced during the extensional tectonic evolution of the Biga Peninsula (Yiğit 2012; Kuşcu et al. 2019a). Thus, the geological setting at Kirazlı and the abundant surface geological and drill hole material offer us the unique opportunity to investigate the detailed history of magmatic and hydrothermal events. In particular, we are interested in understanding whether the PCD and HS epithermal systems belong to a single metallogenic event despite the protracted Eocene to Oligocene magmatic evolution, or represent unrelated, pulsed metallogenic events. The results of our study will have fundamental implications for the genetic interpretation of PCDs-HS epithermal relationships elsewhere in the Biga Peninsula. In this paper, we document the geology and geochemistry of magmatic rocks of the Kirazlı deposit; we present geochronology, including muscovite ^40^Ar/^39^Ar, molybdenite Re-Os, and zircon U-Pb ages, to understand the temporal association of the HS epithermal Au-Ag deposit, the porphyry Cu event, and the Eocene to Oligocene magmatic evolution.

## Regional geology

The Biga Peninsula in northwestern Anatolia is tectonically divided into two entities. The first one is an eastern extension of the Bulgarian-Greek Rhodope Massif, and the second one belongs to the Sakarya zone, which is adjacent to the Intra-Pontide (IPSZ) and Izmir-Ankara-Erzincan Sutures (IAESZ) in the east (Fig. 1A, B). The NE-trending pre-Permian Kazdağ and Permo-Triassic Çamlıca metamorphic rocks form the crystalline basement rocks in the Sakarya and Rhodope zones, respectively (Fig. 1B). The Cretaceous Çetmi ophiolitic mélange in the north-northwest and the Permo-Triassic Karakaya complex comprising of partially metamorphosed volcanic and clastic rocks in the east tectonically overly the crystalline basement rocks (Fig. 1B; Okay and Satir 2000; Bonev and Beccaletto 2007). The pre-Cenozoic rocks are linked to the evolution of the Paleo-Tethys, Intra-Pontide, and the early stages of the Neo-Tethys oceans (Okay et al. 1996; Okay and Satir 2000).

Four main tectonic events controlled the Cenozoic geological evolution: (a) closure of the Neo-Tethys Ocean during northward subduction, (b) Late Cretaceous–early Eocene continental collision of the Anatolide-Tauride Block and the Sakarya zone along the IAES, (c) Eocene to middle Miocene post-collisional tectonics, and (d) middle Miocene to recent subduction of the African plate along the Hellenic Arc beneath the Eurasian plate (Anatolide-Tauride block and Sakarya zone) and tectonic escape (Dewey and Sengor 1979; Sengor et al. 1985), consequently to advanced extension (McKenzie 1978; Pichon and Angelier 1979; Meulenkamp et al. 1988; Harris et al. 1994; Okay and Tuysuz 1999; Dilek and Altunkaynak 2007; Altunkaynak and Genc 2008). Although its timing is still controversial, either Oligocene (Okay and Satir 2000; Bonev et al. 2009) or early-middle Miocene (Cavazza et al. 2009), the extensional deformation contributed to the exhumation of basement rocks along shallow-dipping detachment faults and the emplacement of large plutons at shallow depth (Bozkurt and Park 1997; Bozkurt 2004; Okay and Satir 2000; Kuşcu et al. 2019b).

Cenozoic plutonic rocks in the Biga Peninsula are 52 to 18 Ma old (Yiğit 2012; Kuşcu et al. 2019a). Volcanic rocks in the Biga Peninsula are divided into two groups: (a) pre-Cenozoic mafic volcanic rocks of the Karakaya Complex (Permo-Triassic) and the Çetmi and Denizgören ophiolitic mélanges (Jurassic-Cretaceous), and (b) Cenozoic volcanic rocks hosting numerous precious, base metal, and industrial mineral deposits (Fig. 1B). Cenozoic volcanism produced middle Eocene (Lutetian-Bartonian) medium-K calc-alkaline rocks of the Balıklıçeşme Formation, followed by Oligo-Miocene high-K calc-alkaline to shoshonitic volcanic rocks of Çan and Behram. Finally, the mildly alkaline to alkaline Ezine volcanic rocks were deposited during the late Miocene (Yilmaz 1990; Aldanmaz et al. 2000; Altunkaynak et al. 2012; Yiğit 2012; Kuşcu et al. 2019a). The volcanic rocks become younger towards the southwest and are exposed along the hanging wall of low-angle detachment faults and NE-SW transtensional splays of the North Anatolian Fault Zone (NAFZ; Fig. 1B; Bozkurt 2001; Yiğit 2012; Kuşcu et al. 2019a).

## Geological setting of the Kirazlı deposit

**Fig. 2:**
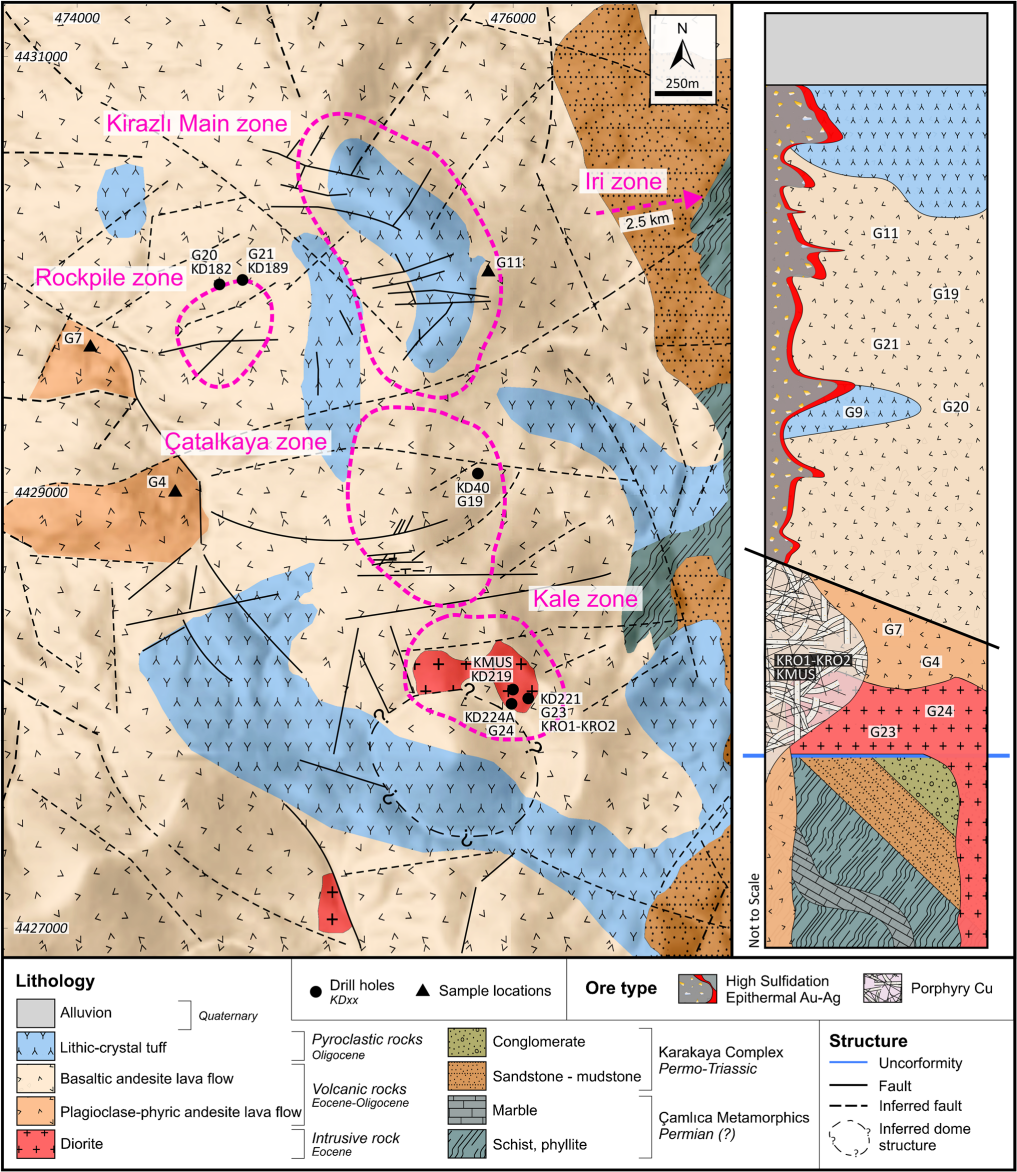
Geological map of the Kirazlı deposit showing lithological and structural elements together with a simplified and synthetic stratigraphic log of the area (the Iri zone is not shown on the map and lies outside of the mapped area, about 2.5 km to the ENE)

**Fig. 3:**
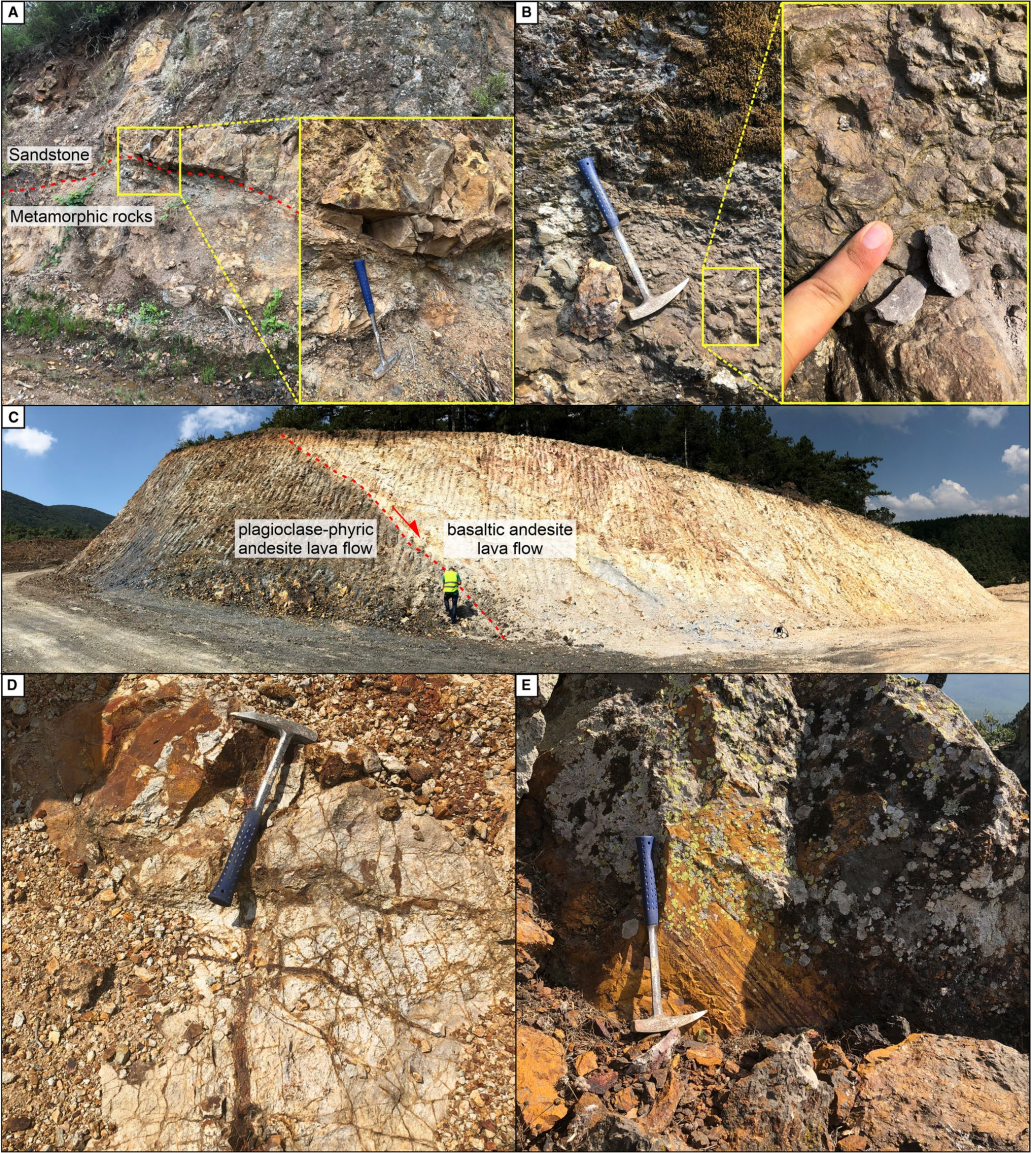
Field relationships of the Kirazlı deposit. (**A**) Contact between basement metamorphic rocks and sandstone-mudstone of the Karakaya complex. (**B**) Conglomerate of the Karakaya complex in SE Kirazlı. Field exposure of (**C**) silicified, brecciated pyroclastic rocks as ledges at the top of Kirazlı Main zone, and (**D**) pervasively altered, stockworked diorite, hosting the porphyry Cu stockwork in the southern Kale zone. (**E**) Boundary between plagioclase-phyric andesite and basaltic andesite

The Permian Çamlıca metamorphic basement rocks within the Kirazlı deposit consist of quartz-mica schist and phyllite with subordinate marble blocks (Fig. 2; Yiğit 2012; Smith et al. 2014; Cormier et al. 2017). They crop out mainly at the eastern margin of the Kirazlı deposit, and are overlain by sandstone, mudstone, and conglomerate of the Permo-Triassic Karakaya Complex (Fig. 3A). The sandstone-mudstone package has been affected by low-grade metamorphism, and consists of locally pyrite-rich arenite, and black shale. The conformably overlying conglomerate includes clasts derived from the basement sandstone and metamorphic rocks (Fig. 3B). The basement rocks are overlain by middle Eocene (Lutetian-Bartonian) to Oligocene diorite, volcanic, and pyroclastic rocks. The Cenozoic rocks are the main host of the Kirazlı HS epithermal and porphyry Cu orebodies (Fig. 2).

### Intrusive rocks

The intrusive rocks are exposed as isolated bodies in the southernmost part of the Kirazlı deposit and have been intersected at shallow levels (~30m) by drill holes in the Kale zone. They are highly altered, which hinders the identification of their original compositional and textural characteristics. The trace element geochemistry suggests an intermediate composition. Therefore, they are named diorite in the remaining part of our study (Figs. 2 and ﻿3D). The automated mineral analyzer (QEMSCAN) has allowed us to identify major minerals (total ratio >80% consisting of primary and secondary minerals) including quartz, plagioclase, and biotite as recognized in an equigranular texture under the microscope. However, the pervasive potassic alteration hinders the identification of the initial mineralogy.

### Volcanic rocks

The Eocene plagioclase-phyric andesite lava flow and Oligocene basaltic andesite lava flow constitute the main volcanic rocks in the Kirazlı deposit (Fig. 2). The plagioclase-phyric andesite is up to 500 m thick and is exposed in the western part of the deposit area. It belongs to the Balıklıçeşme volcanic rocks of the Biga Peninsula based on whole-rock geochemistry and zircon U-Pb geochronology. It is partially altered, including silicification and sericitic alteration. Porphyritic and highly deformed plagioclase dominate the rock with additional magmatic quartz and intensely chloritized subordinate hornblende. Late-stage calcite is pervasive and fills fractures (ESM 2 Fig. S1).

The basaltic andesite dominates in the Kirazlı deposit and unconformably covers the plagioclase-phyric andesite and the diorite (Figs. 2 and 3C). They are part of the regional sequence known as Kirazlı volcanic rocks. They are pervasively altered to clay minerals, partially oxidized, silicified, and chloritized. At deeper levels of the Kirazlı Main zone (~350m), the basaltic andesite lava flow begins with brecciated counterparts, and it transitions into coherent lava flows upward in the section. It is locally intercalated with pyroclastic rocks. The basaltic andesite lava flow consists of unaltered skeletal magmatic quartz with intensely altered plagioclase, hornblende, and pseudocrysts of pyroxene in decreasing order.

### Volcaniclastic rocks

The Oligocene lithic and subordinate crystal tuff are the main volcaniclastic rocks within the Kirazlı deposit. The pyroclastic rocks are locally silicified, oxidized, and brecciated at the topmost levels of the Kirazlı Main zone as well as in the southern part of the Kale zone (Fig. 2). The brecciated rocks crop out as small ledges on the surface (Fig. 3E) and were intersected by drill holes at a vertical depth of up to 400m at the Kirazlı Main zone (Cormier et al. 2017).

### Structural elements

**Fig. 4﻿:**
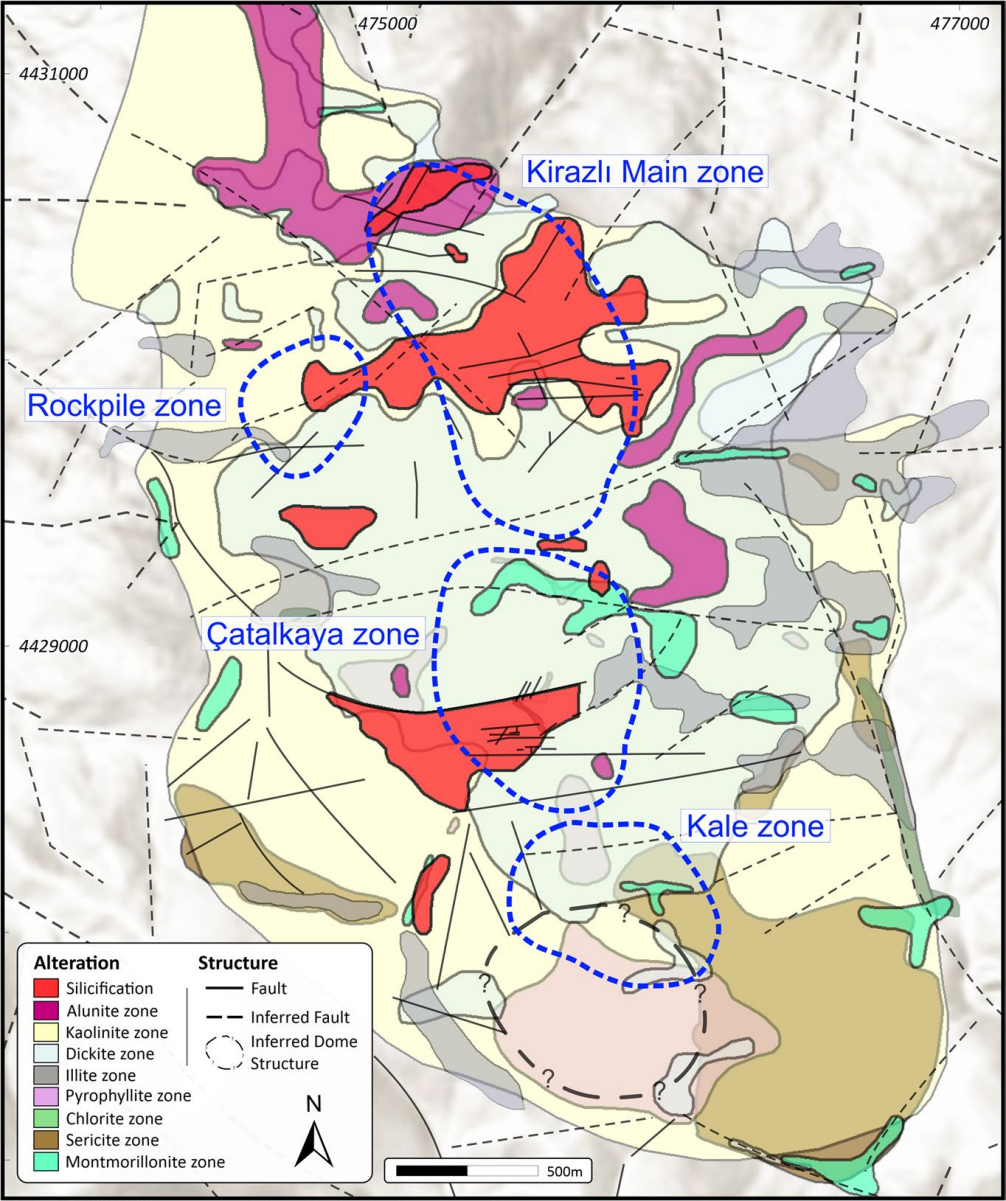
Mineral-based alteration map of the Kirazlı deposit (from Aluç et al. 2023)

The ENE-oriented faults are mainly high-angle normal faults controlling the topographic relief in the Kirazlı deposit, and delineate the morphology of the PCD at the Kale zone and the HS epithermal system at the Kirazlı Main zone (Fig. 2). They form the structural divide between the Kirazlı Main, Çatalkaya, and Kale zones. NW-oriented faults roughly limit the alteration zones in the east and west of the Kirazlı deposit (Figs. 2 and 4). The NNE-oriented faults are sub-vertical, and control the main loci of the high-grade epithermal gold ore bodies, which were later offset by EW-oriented faults in the northern Kirazlı deposit (Cormier et al. 2017).

## The Kirazlı high-sulfidation epithermal Au-Ag deposit and porphyry Cu prospect

The Kirazlı deposit consists of five main mineralized zones, which are from north to south: Kirazlı Main, Rock Pile, Çatalkaya, Kale, and the Iri (Fig. 2, the Iri zone is not shown on the map and lies outside of the mapped area, about 2.5 km to the ENE). Except for the Iri zone, they reflect different vertical levels and horizontal parts of an HS epithermal Au-Ag deposit and a porphyry Cu occurrence. The HS epithermal Au-Ag orebody contains a reserve of 33.86 Mt @ 0.69 g/t Au, 9.42 g/t Ag, and a resource of 3.056 Mt @ 0.43 g/t Au, 2.71 g/t Ag (Alamos Gold 2022). The HS epithermal orebody is hosted by the Oligocene basaltic andesite lava flow and lithic-crystal tuffs. It is NNW-oriented, and is located beneath the Kirazlı Main zone (Fig. 2). The strongly pervasive, widespread, and well-developed hydrothermal alteration zones at the Kirazlı deposit reflect typical patterns of an HS epithermal to porphyry Cu environment towards the Kale zone to the south-southeast (Aluç et al. 2023; Fig. 4). A core-to-rim zoned pattern, from central massive silicification and residual silica, quartz-alunite, a quartz-kaolinite halo, and an outer propylitic alteration envelope, is observed along an E-W-oriented transect at the Kirazlı Main. By contrast, sericitic alteration is more pronounced towards the south at the Kale zone for the porphyry Cu-style environment (Aluç et al. 2023; Fig. 4). Potassic alteration intersected in drill holes at the deeper part of the Kale zone (Aluç et al. 2023).

**Fig. 5:**
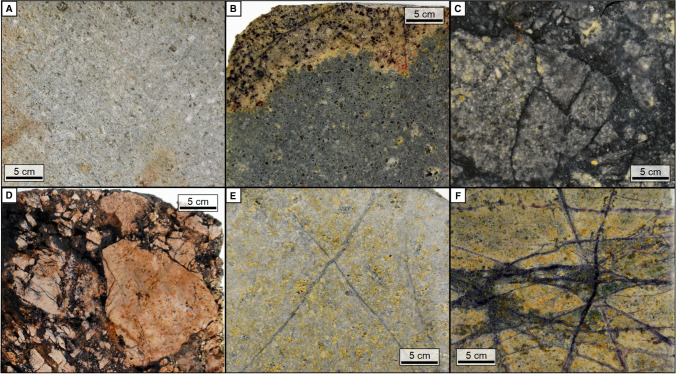
Drill core samples of the basaltic andesite (epithermal host rock). (**A**) Argillic alteration (mainly kaolinite, dickite, and illite) of the basaltic andesite. (**B**) Silicification (mainly residual silica, vuggy textured) and oxidation (hematite) of the basaltic andesite. (**C**) Brecciation of the sulfide zone. (**D**) Brecciation of the oxide zone of the Kirazlı Main zone. (**E**) Millimeter-scale quartz stockwork hosted by sericitic alteration overprinted by argillic alteration in deeper parts of the Kirazlı Main. (**F**) Millimeter-scale quartz stockwork hosted by sericitic alteration overprinted by argillic alteration at shallow depths of the Kale zone

At least two silicification phases and two quartz vein stages have been identified in the HS epithermal mineralization (Cunningham-Dunlop and Lee 2007) including (a) early, barren, and widespread massive silicification and residual silica along the flanks of Kirazlı Main and Çatalkaya zones (Fig. 5B), which is crosscut by (b) yellow-green, sub-horizontal to flat-lying chalcedonic to opaline silica as the second phase representing the silica cap of the system, (c) pyrite- and iron oxide–bearing gray, sub-vertical quartz veins/veinlets filling the fractures of the early phases, in which the silica caps are rooted (Fig. 5C), and (d) late-stage open-space filling crystalline quartz. The sulfide and oxide zones (Fig. 5C and D) of the Kirazlı Main zone host the gold orebodies. The gold ore in the HS system is classified into low- and high-grade (Cormier et al. 2017). The low-grade gold zone is of a regional extent, at the periphery of the high-grade ore zones, and it is hosted by yellow-green chalcedonic to opaline silica. The high-grade gold ore zone is spatially related to the margins of breccia bodies and high-angle silica roots within an argillic zone. The Rock Pile contains local bonanza-gold grades with up to 1080 g/t Au. Gold has been subsequently enriched by supergene oxidation processes affecting the refractory sulfides (Cormier et al. 2017).

**Fig. 6:**
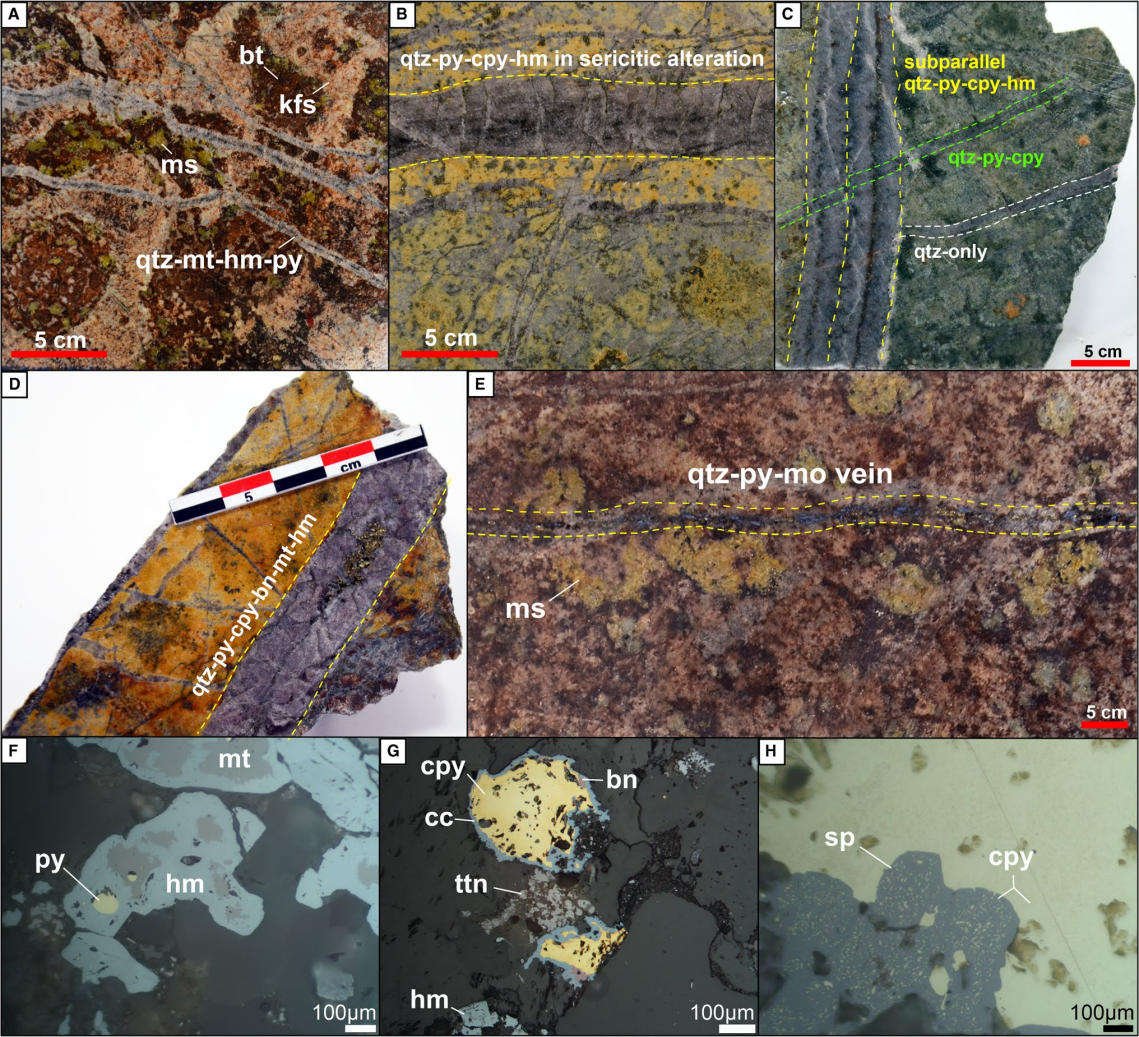
Drill core samples of the porphyry Cu orebody from Kale zone. (**A**) Diorite affected by pervasive potassic alteration overprinted by sericitic alteration. (**B**) Quartz-pyrite-chalcopyrite-hematite (replacing magnetite) vein hosted by a rock with pervasive sericitic alteration overprinting potassic alteration. (**C**) Crosscutting quartz-pyrite-chalcopyrite-hematite veins hosted by chloritic-sericitic alteration. (**D**) Thick quartz vein with pyrite-chalcopyrite and bornite centerline hosted by a rock with pervasive sericitic alteration overprinting potassic alteration. (**E**) Late-stage quartz-pyrite-molybdenite vein crosscutting a rock with potassic alteration and overprinted by sericitic alteration in the Kale zone. (**F**) Hematite replacing magnetite with pyrite. (**G**) Chalcopyrite and bornite replaced by chalcocite alongside hematite (after magnetite) and residual titanite. (**H**) Vein re-opening during the base metal phase: sphalerite with chalcopyrite disease. Abbreviations: bt, biotite; bn, bornite; cc, chalcocite; cpy, chalcopyrite; hm, hematite; kfs, K-feldspar; mo, molybdenite; ms, muscovite; mt, magnetite; py, pyrite; sp, sphalerite; ttn, titanite; qtz, quartz

The porphyry Cu orebody is hosted by the pervasively potassic and sericitic altered Eocene diorites and plagioclase-phyric andesite lava flow in the Kale zone (Fig. 2). The potassic alteration (Fig. 6A) is overprinted by sericitic (Fig. 6B), chloritic (Fig. 6C), and subsequent sodic alteration at depth. The sericitic alteration contains millimeter-scale dark quartz stockworks overprinted by argillic alteration at the surface of the Kale zone (Figs. 4 and 5C and F). The ore zone consists of fine-grained dissemination and subparallel porphyry-style quartz-sulfide veins (Fig. 6B–E), which typically contain bornite, chalcopyrite, pyrite, magnetite, and subordinate molybdenite (Fig. 6D). Thin quartz-pyrite-molybdenite veins crosscut earlier alteration zones and vein systems (Fig. 6E). Hematite replaces primary magnetite (Fig. 6F) alongside the rare precipitation of specularite. Based on drill hole data, the Au, Cu, and Mo concentrations can be as high as 2.49 g/t, 7760 ppm, and 296 ppm, respectively (Cormier et al. 2017). Primary Cu-sulfides were replaced by chalcocite (Fig. 6G) and covellite. Late-stage re-opening of the porphyry veins resulted in the precipitation of a base metal–rich paragenesis, including sphalerite (Fig. 6H) and minor galena.

## Materials and methods

Details of the methodology for each analysis are given at ESM 3. For whole-rock geochemistry, fifteen rock samples from different plutonic and volcanic suites of the Kirazlı deposit were selected. Lithium tetraborate–fused pallets were prepared for each sample and major elements were analyzed by X-ray fluorescence (XRF) and trace elements, including rare earth elements (REE), were determined using an Element XR sector-field ICP-MS at the University of Lausanne. Three ablation spots were collected on each sample and the data reduction processes were performed using LAMTRACE (Jackson 2008).

Traditional mineral separation techniques (crushing, milling, shaking table, magnetic separation, and heavy liquid) were applied to extract zircon grains from nine samples for U-Pb geochronology. Before LA-ICP-MS analyses, cathodoluminescence (CL) images were taken to define zoning and select the location of the laser spots, using a CamScan MV 2300 SEM at the University of Geneva. Dating was performed using the same ICP-MS and ablation system for the analysis of lithium tetraborate pellets at the University of Lausanne. Raw data were processed offline using the LAMTRACE software (Jackson 2008). The weighted mean age diagrams showing the ages calculated from U-Pb isotope ratios and U-Pb frequency plots were generated using the Isoplot/Ex v. 4.15 software (Ludwig 2012) after excluding discordant and outlier data.

Two molybdenite samples were extracted from quartz-pyrite-molybdenite veins (Fig. 6E and H) within sericite-rich alteration assemblages in the Kale zone under binocular microscope at the Muğla Sıtkı Koçman University for Re-Os dating. Detailed sample preparation and analytical protocols are documented by Selby and Creaser (2001, 2004), Selby et al. (2007), and Lawley and Selby (2012). The ^187^Re and ^187^Os isotope ratios were determined using isotope-dilution Negative Thermal Ionization Mass Spectrometry on a Thermo Scientific TRITON mass spectrometer using static Faraday collection at the University of Durham (UK). The Re-Os dates are calculated using ^187^Re decay constants from Smoliar et al. (1996).

For ^40^Ar/^39^Ar dating, the muscovite aliquot selected from potassic alteration overprinted by sericitic alteration at the Kale zone was weighed, wrapped in Cu foil, and placed between Fish Canyon Tuff (FCT) sanidine flux monitors in a linear stack in quartz tubes, and irradiated for 15 h in the Oregon State University TRIGA reactor using the shielded CLICIT site. The step-heating experiment included 14 heating steps, with a blank measurement after each analysis step. Argon isotopes were measured on a multi-collector Thermo Scientific Argus VI mass spectrometer in static mode at the University of Geneva. All data regression was done using ArArCalc (Koppers 2002).

## Results

### Whole-rock geochemistry

**Fig. 7:**
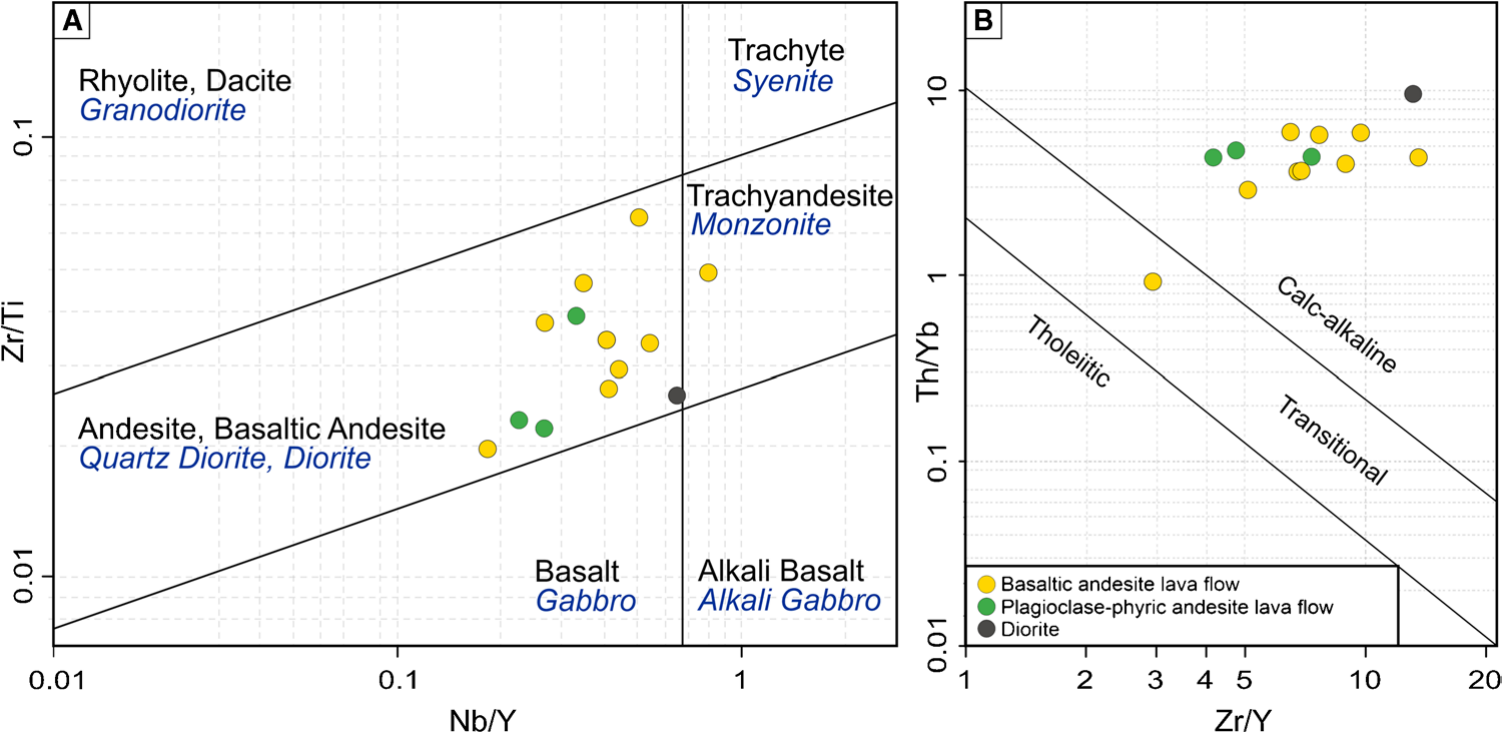
(**A**) Zr/Ti vs Nb/Y classification diagram of volcanic rocks (Winchester and Floyd 1977). (**B**) Th/Yb vs Zr/Y discrimination diagram (Ross and Bédard 2009)

The whole-rock geochemistry results of fifteen samples are shown in ESM 1 Table S2. The high field strength elements (HFSE) and rare earth elements (REE) have been mainly used for classification and interpretation purposes since they are less affected by hydrothermal alteration (Rollinson 1993). The Zr/Ti vs. Nb/Y discrimination diagram (Pearce 1996) was used to name volcanic and plutonic rocks (Fig. 7A). The Zr/Y vs Th-Yb discrimination diagram (Hastie et al. ﻿^2007^) shows that volcanic and plutonic rocks from the Kirazlı deposit have a dominantly calc-alkaline character (Fig. 7B). Petrographic studies of basaltic andesite and plagioclase-phyric andesite are in good agreement with discrimination diagrams. Although pervasive alteration almost obliterated the original mineral paragenesis of the intrusive rocks from the Kale zone, Fig. 7A indicates a dioritic/quartz dioritic composition for one sample (G24 in ESM ﻿1 Table S2).

**Fig. 8:**
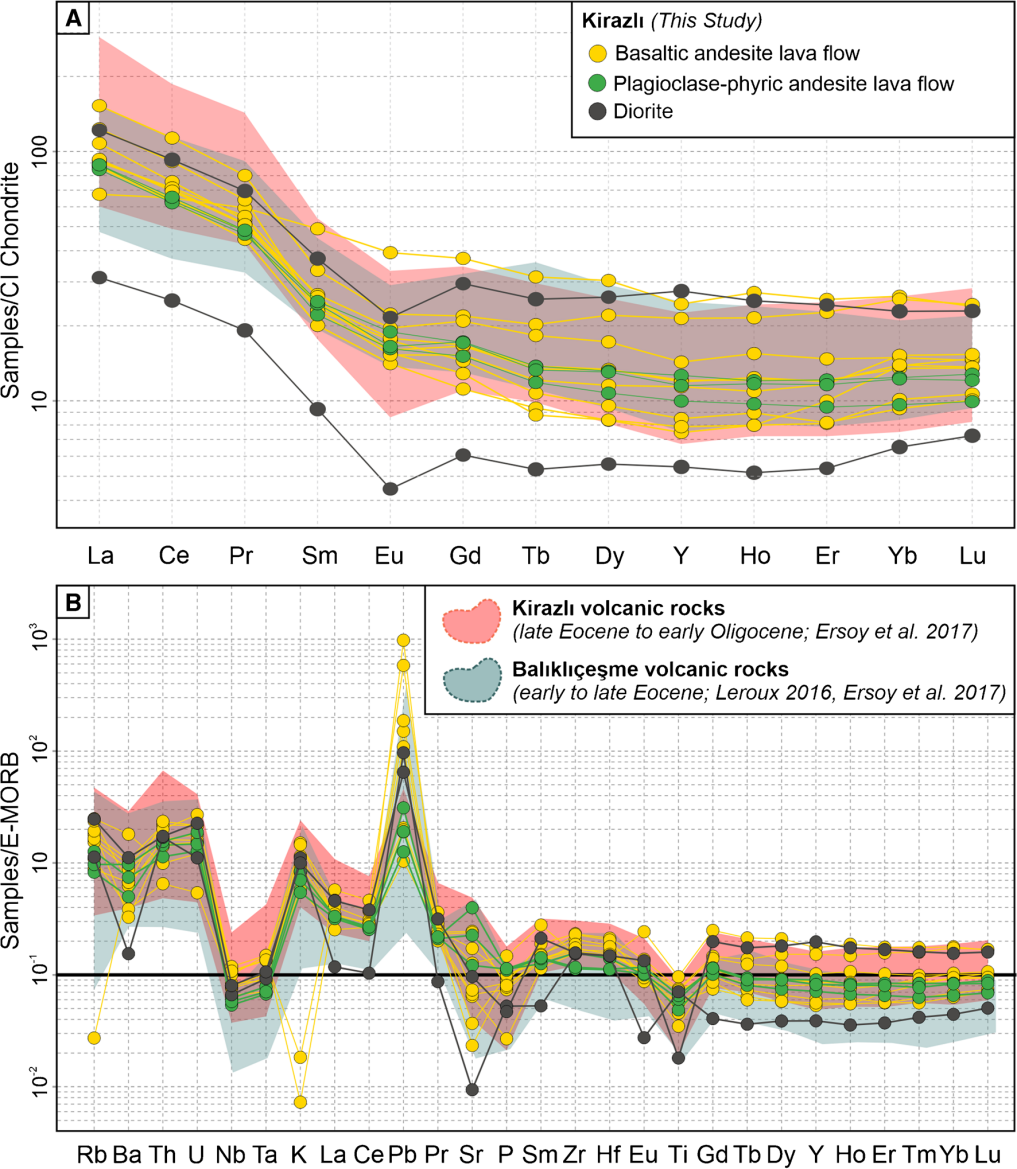
(**A**) Chondrite-normalized REE patterns (Sun and McDonough 1989) and (**B**) E-MORB-normalized multi-element patterns (McDonough and Sun ﻿^1995^) for samples from the Kirazlı deposit (Kirazlı and Balıklıçeşme data from Leroux 2016 and Ersoy et al. 2017, respectively)

The CI chondrite–normalized rare earth elements and E-MORB-normalized trace elements patterns of volcanic and plutonic rocks from the Kirazlı deposit area are shown in Fig. 8A and B, respectively. The geochemical data for the regional Kirazlı and Balıklıçeşme volcanic rocks (Leroux 2016; Ersoy et al. 2017) are presented on the same diagrams for comparison purposes. The intrusive rocks from the Kale zone show a similar trace element pattern despite different values due to the degree of alteration. All samples exhibit a humped-back pattern in the CI chondrite–normalized REE plot (Fig. 8A). Most of the volcanic rocks show slight negative Eu anomalies, whereas the intrusive rocks at the Kale zone have a strong negative Eu anomaly (Fig. 8A). All samples from the Kirazlı deposit have negative Nb and Ta anomalies and enrichment in large ion lithophile (LIL; K, Rb, Th, and Ba) elements relative to HFS elements except two outliers from the basaltic andesite (Fig. 8B).

### Zircon U-Pb geochronology

**Fig. 9:**
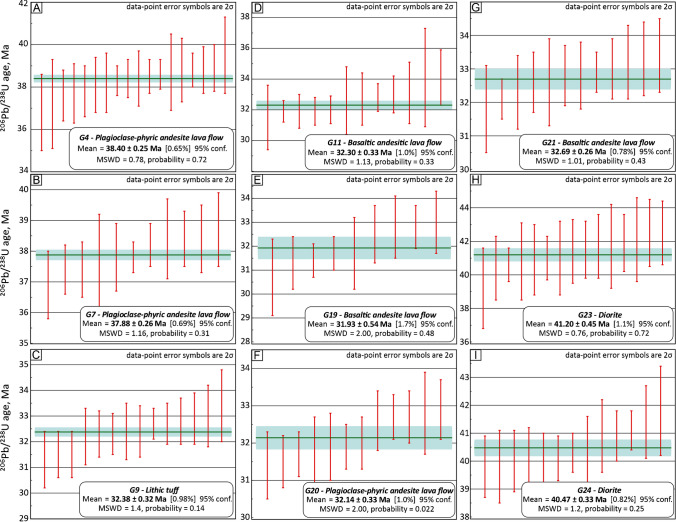
Weighted mean average diagrams of ^206^Pb/^238^U ages of zircons from the Kirazlı epithermal and porphyry Cu systems

The results of the LA-ICP-MS U-Pb zircon geochronology from the Kirazlı deposit are summarized in Table 1. The weighted average mean diagrams are shown in Fig. 9. The average mean ages for the diorite from the Kale zone are 40.5 ± 0.3 and 41.2 ± 0.5 Ma, for plagioclase-phyric andesite 37.9 ± 0.3 and 38.4 ± 0.3 Ma, between 31.9 ± 0.5 and 32.7 ± 0.3 Ma for basaltic andesite and 32.4 ± 0.3 Ma for lithic tuff in the Kirazlı deposit. These ages show three different age suites; one for the plutonic activity during the middle Eocene (Lutetian-Bartonian), and two ages for the volcanism in the late Eocene (Bartonian) and the early Oligocene (Rupelian).

**Table 1:** U-Pb zircon geochronology results for the Kirazli epithermal and porphyry Cu systems and the surrounding region

Sample	Location								Rock type		Age (Ma)^3^ ± 2σ	MSWD^4^	*n*	*n*^5^
	Prospect	Zone	Deposit^1^	Easting	Northing	Drill Hole	From (m)	To (m)	Lithology^2^	Unit				
G4	Kirazlı	Main	HS	474467	4428995				PAnd	Host	38.4 ± 0.3	0.78	26	18
G7	Kirazlı	Main	HS	474172	4429821				PAnd	Host	37.9 ± 0.3	1.16	19	11
G9	Kirazlı	Main	HS	472678	4431418				LiTuff	Host	32.4 ± 0.3	1.4	32	14
G11	Kirazlı	Main	HS	475928	4430256				BAnd	Host	32.3 ± 0.3	1.13	34	12
G19	Kirazlı	Çatalkaya	HS	475710	4428793	KD40	137.00	148.00	BAnd	Host	31.9 ± 0.5	2.0	27	9
G20	Kirazlı	Rock Pile	HS	474712	4429946	KD189	145.00	150.00	BAnd	Host	32.1 ± 0.3	2.0	21	12
G21	Kirazlı	Rock Pile	HS	474630	4429933	KD182	90.70	98.70	BAnd	Host	32.7 ± 0.3	1.01	18	12
G23	Kirazlı	Kale	PCD	476111	4428309	KD221	214.00	220.00	Diorite	Host	41.2 ± 0.5	0.76	24	15
G24	Kirazlı	Kale	PCD	475994	4428319	KD224A	50.00	58.00	Diorite	Host	40.5 ± 0.3	1.2	28	13

^1^*HS*, high sulfidation; *PCD*, porphyry Cu

^2^*BAnd*, basaltic andesite, *PAnd*, plagioclase-phyric andesite, *LiTuff*, lithic tuff

^3^Weighted mean average age

^4^Mean square of weighted deviates

^5^Excluding discordant and outlier LA-ICP-MS analyses (lower row) and antecrystic zircons (upper row)

### Molybdenite Re-Os geochronology

The Re-Os geochronology results of two molybdenite samples from the Kale zone porphyry Cu mineralization are presented in Table 2. Sample KRO1 was collected from a 0.2- to 1-cm-thick quartz-pyrite-molybdenite vein, and sample KRO2 was collected from disseminated molybdenite in the sericite-rich alteration zone. The total Re concentrations are 7657 and 6323 ppm, respectively, and ^187^Os concentrations are 2696 and 2229 ppb. Samples KRO1 and KRO2 yield Re-Os ages of 33.6 ± 0.2 and 33.7 ± 0.2 Ma, respectively (Table 2). Therefore, the molybdenite event took place in the early Oligocene (Rupelian).

**Table 2:** Molybdenite Re-Os data for the Kirazlı porphyry Cu system

Sample no	Drill hole	From (m)	To (m)	wt (g)	Re (ppm) ± 2σ	^187^Re (ppm) ± 2σ	^187^Os (ppb) ± 2σ	Age (Ma) ± 2σ (1)	Age (Ma) ± 2σ (2)
KRO1	﻿KDD221	29.60	29.70	0.0118	7657.2 ± 35.4	4812.7 ± 22.3	2696.0 ± 11.3	33.6 ± 0.1	33.6 ± 0.2
KRO2				0.0108	6323.1 ± 30.7	3974.2 ± 19.3	2228.5 ± 10.0	33.7 ± 0.1	33.7 ± 0.2

Re-Os dates are calculated using ^187^Re decay constants from Smoliar et al. (1996)

(1) Age uncertainty includes all sources of analytical uncertainty

(2) Age uncertainty includes all sources of analytical uncertainty and that of the decay constant

### Muscovite ^40^Ar/^39^Ar geochronology

**Fig. 10:**
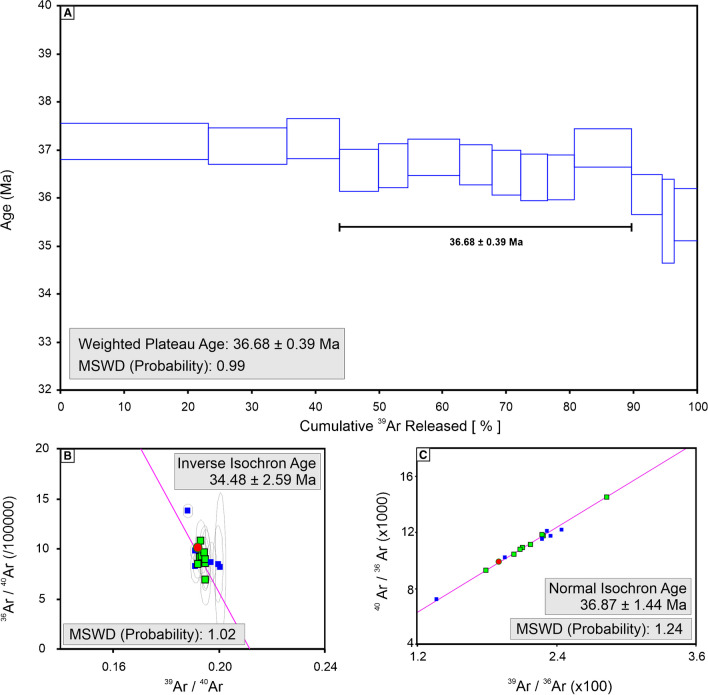
^40^Ar/^39^Ar (**A**) plateau, (**B**) normal isochron, and (**C**) inverse isochron age plots of the muscovite (KMUS) sample from sericitic alteration of the Kirazlı porphyry Cu orebody

The analytical results with errors at the 2σ confidence interval for muscovite (KMUS) separate, collected from the sericite-rich alteration at the Kale zone, are presented in Table 3. A reliable age is estimated using the plateau diagram with eight contiguous heating steps (GE110-15 to GE110-23). The plateau yields an age of 36.7 ± 0.4 Ma with an MSWD of 0.99 (Fig. 10A). The normal isochron age is 36.9 ± 1.4 Ma (Fig. 10B), and the inverse isochron age is 34.5 ± 2.6 Ma (Fig. 10C), which are consistent with the plateau age.

**Table 3:** ^40^Ar/^39^Ar data for the muscovite sample (KMUS) from the Kirazlı porphyry Cu system

Sample	Drill hole	From (m)	To (m)	Step No	^36^Ar(a) [fA]	^37^Ar(ca) [fA]	^38^Ar(cl) [fA]	^39^Ar(k) [fA]	^40^Ar(r) [fA]	Age (Ma) ± 2σ
﻿KMUS	KD219	81.70	81.80	KMUS-GE110-15	0.1129071	0.926631	0.1224446	202.5714	1014.935	36.6 ± 0.4
				KMUS-GE110-16	0.0545120	0.732150	0.0411086	154.3771	775.585	36.7 ± 0.5
				KMUS-GE110-17	0.1267864	1.310175	0.1531105	267.0976	1348.354	36.9 ± 0.4
				KMUS-GE110-19	0.0804279	0.972384	0.0554925	167.9719	844.206	36.7 ± 0.4
				KMUS-GE110-20	0.0642816	0.911588	0.0799789	147.0524	735.793	36.5 ± 0.5
				KMUS-GE110-21	0.0643770	0.890030	0.0872036	140.0715	698.848	36.4 ± 0.5
				KMUS-GE110-22	0.0677611	0.916845	0.0941793	137.8756	687.978	36.4 ± 0.5
				KMUS-GE110-23	0.1298585	1.757464	0.1158651	295.5895	1499.994	37.0 ± 0.4
									Plateau age:	36.7 ± 0.4

## Discussion

### Tectonic setting and source characteristics of magmatic rocks

Cenozoic volcanism in the Biga Peninsula has been active from the late Paleocene (~*52 Ma*) to early-middle Miocene (*~18 Ma*) driven by three main tectonic events accompanied by episodic extensional tectonic phases (Yiğit 2012; Sánchez et al. 2016; Kuşcu et al. 2019a). These events are (a) the collision of the Anatolide-Tauride Block and Sakarya zone along the IAES zone, followed by (b) post-collisional settings, and (c) subduction along the Hellenic Arc with subsequent extension and core complex exhumation. These events resulted in a change in the geochemical composition of the magmatic rocks through time with different tectonic episodes and sub-episodes (Kuşcu et al. 2019b). The three main magmatic phases are (1) middle Eocene (Lutetian-Bartonian) to Early Miocene, mainly calc-alkaline and high-K subduction-related magmas, (2) early to middle Miocene shoshonitic and crustal contaminated magmas, and finally (3) late Miocene sodic back-arc basalts (Agostini et al. 2010). Geochemical studies suggest a decreasing subduction component and increasing crustal contamination from the Eocene to the early Miocene (Altunkaynak and Genc 2008).

The host rocks and adjacent rock units of the HS and porphyry Cu orebodies at the Kirazlı deposit show a calc-alkaline affinity (Fig 7B). The trace element data for these rocks, as well as of similar rock units of the same period (e.g., Balıklıçeşme Formation, and Kirazlı volcanic rocks; Leroux 2016; Ersoy et al. 2017), were compared in multi-element diagrams (Fig. 8). The enrichment in LILE (Ba, Cs, Rb, Th, U) and depletion in HFSE (Nb, Ta, Y, Zr, Ti) can be attributed to enrichment in the mantle source (metasomatized mantle in a subduction setting) and/or crustal contamination (Fig. 8B). In the multi-element E-MORB-normalized diagram (Sun and McDonough 1989), there is a clear enrichment in Rb, U, and Th, and depletion in Nb, Ta, and Ti in all units, which indicate melting of a subduction-modified metasomatized mantle at a convergent margin setting (Fig. 8B). The negative Eu anomalies favor plagioclase fractionation and/or an oxidized nature of the magmas, which are getting less pronounced from the middle Eocene (Lutetian-Bartonian) diorite to the Oligocene basaltic andesite as suggested by Kuşcu et al. (2019a).

**Fig. 11:**
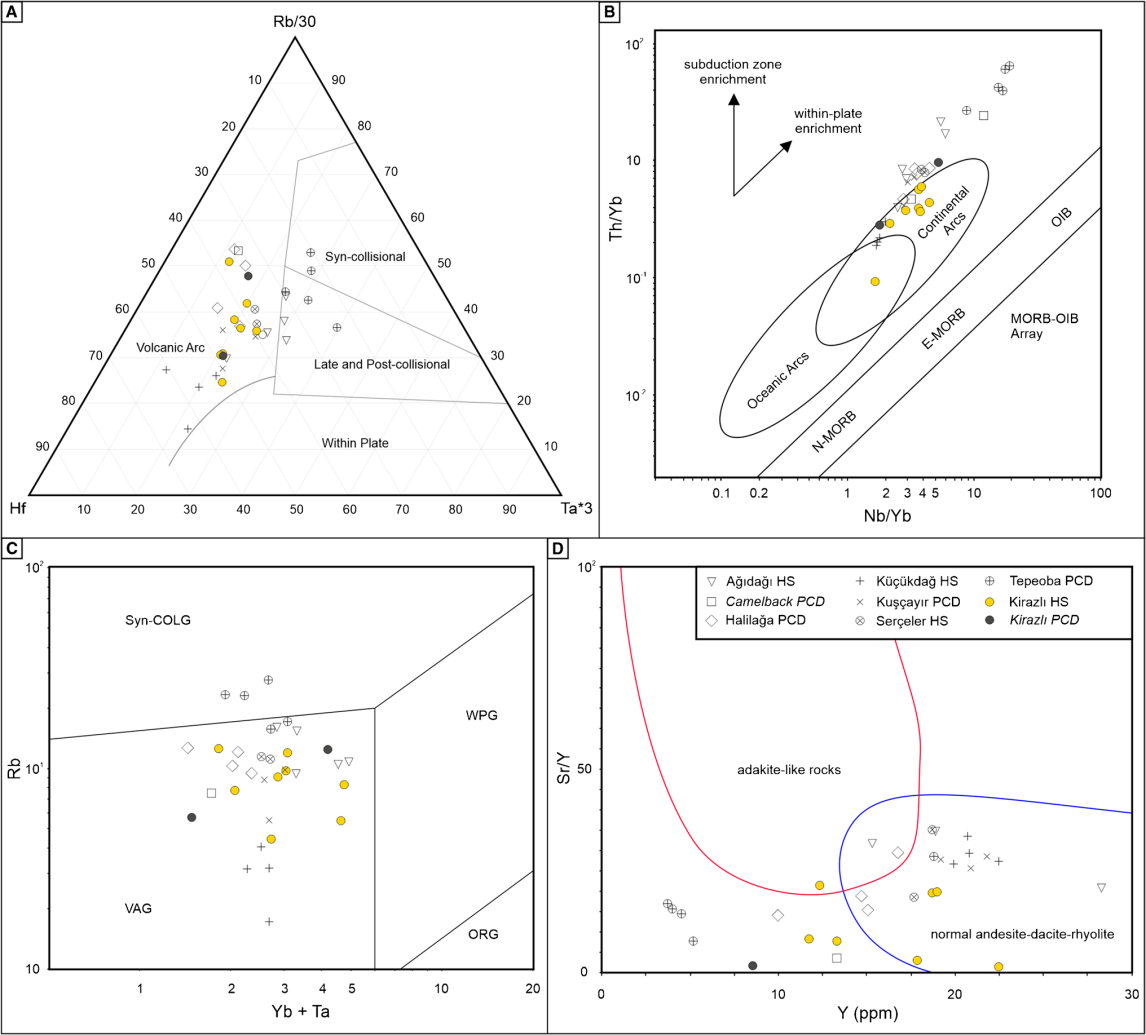
(**A**) Rb/30-Hf–Ta*3 tectonic discrimination diagram (Harris et al. 1994); (**B**) Th/Yb vs Nb/Yb discrimination diagram, (MORB - Mid-Oceanic Ridge Basalt, OIB - Ocean-Island Basalt, N-MORB - Normal-MORB, E-MORB - Plume-MORB; Pearce 2014); (**C**) Rb vs Yb + Ta tectonic setting discrimination diagram (Pearce et al. 1984); (**D**) Sr/Y vs Y discrimination diagram for adakite-like and non-adakitic rocks (fields for adakite-like rocks and normal-arc andesite-dacite-rhyolite are from Richards et al. ﻿^2012^)

The geochemical data of the Kirazlı host rocks and selected rocks from other deposits were used for a better understanding of the tectonic evolution of the Biga Peninsula. The Hf – Rb/30 – Ta*3 discrimination diagram (Rollinson 1993) indicates a typical volcanic arc-setting (Fig. 11A), despite the outlier geochemistry of the late Oligocene systems (e.g., Tepeoba porphyry Cu – Mo, Ağıdağı HS deposits). The Th/Yb vs. Nb/Yb and the Rb vs. Yb+Ta diagrams also support a volcanic arc geochemical signature (Fig. 11B, C) except for some of the data from the late Oligocene systems, which show a within-plate enrichment trend in Fig. 11B (Pearce et al. 1984; Pearce 2014). This demonstrates an increasing assimilation-fractional crystallization (AFC) and/or crustal contamination from the middle Eocene (Lutetian-Bartonian) towards the late Oligocene as suggested in Altunkaynak and Genc (2008). In addition, the Sr/Y vs. Y diagram indicates a normal-arc andesite-dacite affinity (Fig. 11D).

**Fig. 12:**
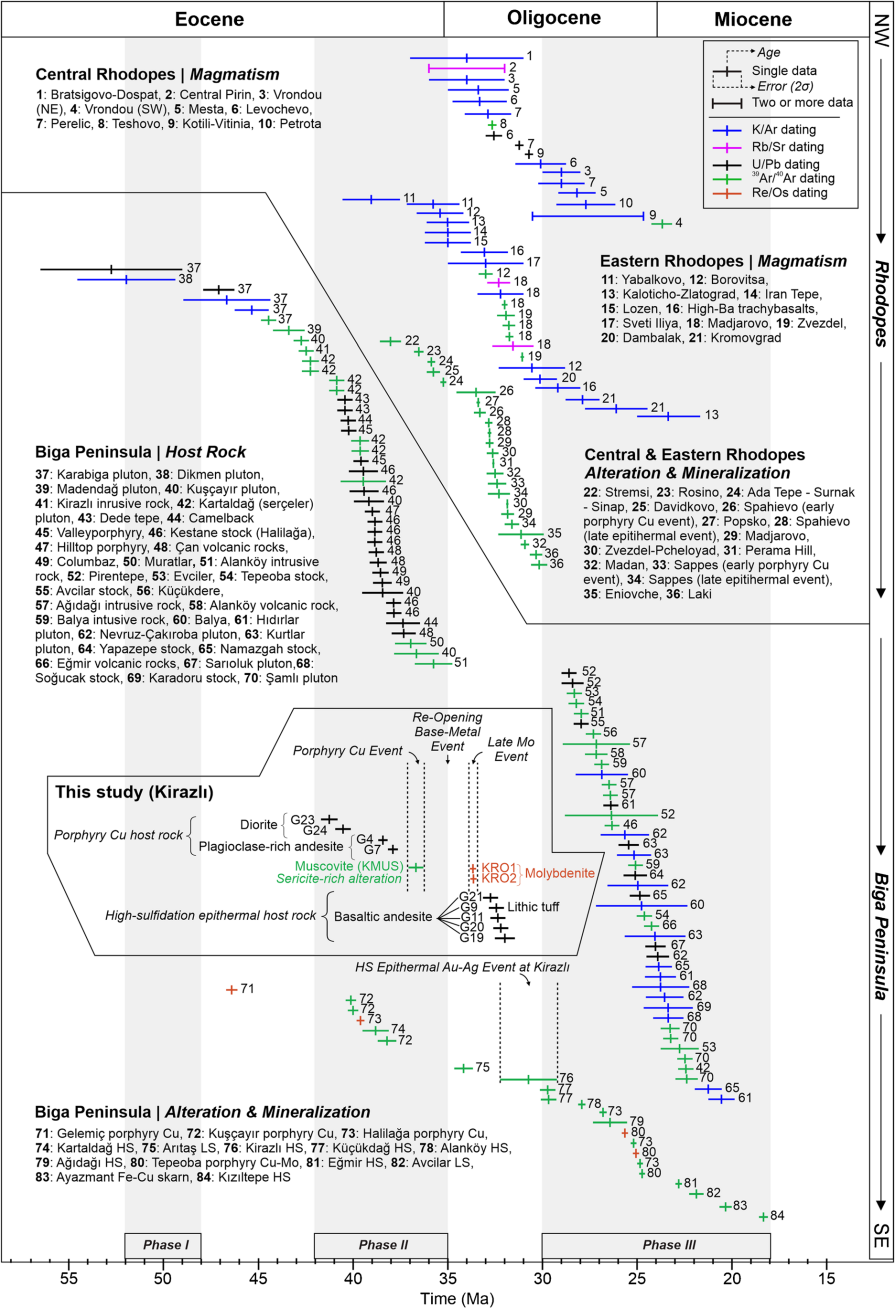
Isotopic ages of magmatic rocks, and alteration and mineralization events of the Biga Peninsula and the Rhodope Massif. The shaded areas depicting magmatic Phases I, II, and III refer to ore-related episodes defined by Kuşcu et al. (2019b). All radiometric data together with corresponding references are given in ESM 1

The rock units at the Kirazlı deposit have been dated between middle Eocene (*41.2 ± 0.5 Ma*) and early Oligocene (*31.9 ± 0.5 Ma*) (Fig. 12 and ESM 2 Fig. S2) suggesting that they formed during post-collisional tectonic and magmatic evolution. Considering the REE, multi-element, and tectonic discrimination diagrams, we conclude that the volcanic and plutonic rocks of the Kirazlı deposit were sourced from a subduction-modified metasomatized mantle, and were affected by AFC processes and/or crustal contamination.

### Evolution and timing of magmatism and hydrothermal activity at the Kirazlı deposit

LA-ICP-MS zircon U-Pb geochronology of the basaltic andesite and lithic/crystal tuff, which are the host rocks of the HS epithermal deposit collected at different depths, yielded an age between 31.9 ± 0.5 and 32.7 ± 0.3 Ma. Yiğit (2012) reported a ^40^Ar/^39^Ar age of 30.7 ± 1.5 Ma for an alunite-rich whole-rock sample containing 50% alunite and 50% quartz from the HS environment of the Kirazlı Main zone, which is coherent with its host rock age. It indicates that the HS epithermal Au-Ag mineralization is related to the Oligocene magmatism in the Kirazlı deposit.

The LA-ICP-MS zircon U-Pb geochronology of the four samples from the host rocks of the porphyry Cu mineralization yielded ages ranging from 37.9 ± 0.3 to 38.4 ± 0.3 Ma (for plagioclase-phyric andesite) and from 40.5 ± 0.3 to 41.2 ± 0.5 Ma (for the diorite). These ages are indicative of different magmatic pulses during each mineralization stage in the Kirazlı deposit. Our data indicate that the host rock of the HS epithermal orebody is younger than at the porphyry Cu orebody.

The ^40^Ar/^39^Ar age of muscovite from the sericite-rich alteration assemblage in the porphyry Cu ore zone yielded an age of 36.7 ± 0.4 Ma, which postdates the LA-ICP-MS zircon U-Pb ages of its host rocks. Therefore, the porphyry Cu mineralization event is significantly younger than the Eocene diorite (Fig. 12, and ESM 2 Fig. S2). The 33.6 ± 0.2 and 33.7 ± 0.2 Ma Re-Os ages yielded by the two molybdenite samples from the latest quartz-molybdenite-pyrite vein stage crosscutting all previous alteration and mineralization assemblages. The Re-Os ages indicate that the quartz-pyrite-molybdenite veins and disseminated molybdenite postdate the porphyry Cu orebody by about 3 Myr.

Previous geological studies within and nearby the Kirazlı deposit have demonstrated that the Biga Peninsula has been under regional-scale extensional tectonics, which has resulted in the exhumation of the metamorphic basement along detachment faults and tectonic displacement of the ore deposits (Kissel et al. 1987; Bonev and Beccaletto 2007; Cavazza et al. 2009; Jolivet and Brun 2010; Yiğit 2012; Sánchez et al. 2016). The detachment faults together with NE-oriented normal and strike-slip faults controlled the emplacement of younger magmatic events and younger hydrothermal systems overprinting older mineralized systems within the same area. Sánchez et al. (2016) state that many hydrothermal systems in the Biga province were tilted to the north in response to the Cenozoic deformation accommodated by SSE-dipping extensional faults generating block rotations as in arigid domino system.

The geochronological data presented here also indicate that the HS epithermal system in the Kirazlı deposit is much younger than, and genetically unrelated, to the spatially associated Kale porphyry Cu event (Fig. 12, and ESM 2 Fig. S2). Therefore, this excludes a temporal and genetic link with the same magmatic-hydrothermal system of both ore systems in the Kirazlı deposit. Indeed, the typical life span of telescoped porphyry-epithermal systems ranges from 1 Ma to <300.000 years (Muntean and Einaudi 2001; Masterman et al. 2005).

The new radiometric ages together with comprehensive geological investigation suggest that the porphyry Cu orebody formed in the late Eocene and has been subsequently tilted/uplifted as a consequence of regional tectonics. In addition, early quartz veins that formed at high temperatures in the porphyry Cu ore zone were reopened and filled by different generation(s) of quartz and sulfides at lower temperatures in the Kirazlı deposit (Fig. 12, and ESM 2 Fig. S2; Aluç et al. 2021). This is analogous to post-ore re-opening described in other ore deposits, such as at Bingham Canyon (Redmond and Einaudi 2010), Santa Rita (Tsuruoka et al. 2021), and El Salvador (Watanabe et al. 2018). Considering geological and geochronological constraints, we conclude that the late Oligocene HS epithermal system overprinted the porphyry Cu orebody at shallow depth, and thus resulted in genetically disconnected epithermal and porphyry systems at Kirazlı. Further structural studies in the Kirazlı deposit may clarify the exact tectonic evolution between porphyry Cu ore formation and HS epithermal emplacement.

### Regional temporal and spatial correlation of the Kirazlı epithermal and porphyry systems with the Biga Peninsula and the Rhodope Massif

Cenozoic magmatism and associated mineralization in the Biga Peninsula become younger towards the south except for one outlier whole-rock K-Ar age of granite from the central-east located Yenice pluton (18.8 ± 1.3 Ma; Karacik et al. 2008). In the Biga Peninsula, because of the absence of reliable alteration and mineralization isotope ages, the hydrothermal episodes were defined using solely host rock radiometric ages. Previously, the magmatic episodes related to known ore deposits in the Biga Peninsula have been divided into three main phases by Kuşcu et al. (2019b), including collision-related magmatism (Phase I at 52–48 Ma), post-collisional magmatism (Phase II at 42–35 Ma), and a subduction coeval with extension-related magmatism (Phase III at 30–18 Ma, Fig. 12). Most of the products of these three magmatic phases have a high-K calc-alkaline affinity. The earliest magmatic Phase I is associated with a limited number of deposits and occurrences in the northern Biga Peninsula (Fig. 1B; e.g., Dikmen porphyry Mo-Cu±Au mineralization, 51.9 ± 2.6 to 46.6 ± 2.3 Ma; whole-rock K/Ar; Yiğit 2012). The second Phase II (42 to 35 Ma) includes widespread volcanism and plutonism in the central part of the peninsula (e.g., Eocene Kuşçayır, Kapıdağ plutons, and Yeniköy stock), and is associated with several porphyry-epithermal Au-Ag-Cu deposits, including the Halilağa porphyry Cu (39.6 ± 0.21 Ma; molybdenite Re-Os; Brunetti 2016), Kartaldağ HS Au (38.8 ± 0.7 Ma; alunite-quartz-rich whole-rock ^40^Ar/^39^Ar; Yiğit 2012), and the Valley porphyry Cu-Au (Kuscayir district; 40.2 ± 0.4 Ma; zircon U-Pb; Smith et al. 2016) deposits, which are typical examples of the Phase II. The youngest Phase III (30–18 Ma) is associated with more abundant hydrothermal centers than the earlier magmatic phases. The Ağıdağı HS Au-Ag (25.8 ± 1.4 Ma; alunite (70%) + quartz (30%) whole-rock ^40^Ar/^39^Ar; Yiğit 2012), Tepeoba porphyry Cu – Mo (24.6 ± 0.2 Ma; molybdenite Re-Os; Murakami et al. 2005), and Küçükdağ HS Au-Ag-Cu (29.2 ± 0.3 Ma; alunite ^40^Ar/^39^Ar; Leroux 2016) deposits are typical examples of the latest ore-forming phase.

In the Bulgarian and Greek Rhodope Massif, deposits and prospects are hosted by high-grade metamorphic, continental sedimentary, and igneous rocks (Marchev et al. 2005) and commonly formed contemporaneously together with their causative magmatism (Bonev et al. 2013; Moritz et al. 2014). Single-continuous hydrothermal magmatic episode associated with extensional exhumation together with doming and late faulting took place between *ca.* 39 and 30 Ma (Fig. 12). The oldest mineralization event is recognized at the Stremtsi gold prospect dated at 37.6±0.3 Ma (Moritz et al. 2014) followed by epithermal prospects at Ada Tepe and Rosino dated at 35.4 ± 0.2 Ma (Marton et al. 2010) and 36.5 ± 0.3 Ma (Bonev et al. 2006), respectively. The economically most important deposits (such as Laki, Davidkovo, Ardino, Madan, and Thermes ore fields in the Central Rhodopes, and Popsko in the Eastern Rhodopes) have been formed between *ca.* 33 and 30 Ma (Fig. 12, Marchev et al. 2005; Bonev et al. 2013; Moritz et al. 2014).

The alteration ages of the Kirazlı HS epithermal and porphyry Cu systems are 30.7 ± 1.5 Ma (Yiğit 2012) and 36.7 ± 0.4 Ma, respectively. The later age correlates with the mineralization events that took place in the late stages of magmatic Phase II of Kuşcu et al. (2019b) at 42 to 35 Ma, whereas the HS epithermal Au-Ag event belongs to the onset of the latest magmatic Phase III of Kuşcu et al. (2019b) at 30–18 Ma. Our radiometric ages from the mineralization and host rocks allow us to define a detailed temporal and spatial framework of the Kirazlı deposit. The zircon U-Pb age of the porphyry Cu host rocks is *ca.* 38–41 Ma and overlaps with other intrusions at nearby districts (e.g., Halilağa, Kuşçayır). Yet, the age of mineralization is *ca.* 36 Ma and was followed by base metal precipitation during the re-opening of the porphyry Cu veins. Thereafter, late molybdenite crosscuts the previous events at *ca.* 33 Ma, in between the ore-forming Phases II and III defined by Kuşcu et al. (2019b); Fig. 12). The zircon U-Pb age of the HS epithermal basaltic andesitic host rock is *ca.* 32, which fills the gap between Phases II and III of Kuşcu et al. (2019b); (Fig. 12).

The Kirazlı deposit is not the only outlier in the scheme defined by Kuşcu et al. (2019b). Adularia ^40^Ar/^39^Ar dating of the Arıtaş low sulfidation system in the southern Biga Peninsula yielded an age of 34.1 ± 0.5 Ma (Fig. 12, Sánchez et al. 2016) and falls also between Phases II and III of Kuşcu et al. (2019b).

Our study at Kirazlı reveals intermittent phases of ore formation between regional hydrothermal ore-forming Phases II to III defined in earlier studies (Kuşcu et al. 2019b). Therefore, we suggest that the post-collisional Phase II magmatism should be extended between 42 and 30 Ma. This indicates that there is no magmatic-hydrothermal lull in Biga Peninsula after middle Eocene (Lutetian-Bartonian) like in the Rhodope Massif. We expect that future studies with an increasing number of precise radiometric ages of hydrothermal alteration, mineralization, and magmatic events will certainly support our interpretation.

## Conclusions

We have demonstrated the Kirazlı mineral deposit hosts spatially associated but genetically unrelated HS epithermal Au-Ag and porphyry Cu orebodies. The magmatic-hydrothermal evolution of the Kirazlı deposit has recorded *ca.* 10 My-long evolution with episodic magmatic and hydrothermal events. Geology, geochemistry, and zircon U-Pb geochronology reveal that there have been three phases of calc-alkaline magmatism, including (1) early diorite intrusion at 40.5 ± 0.3 to 41.2 ± 0.5 Ma, (2) plagioclase-phyric andesite at 37.9 ± 0.3 to 38.4 ± 0.3 Ma, and (3) basaltic andesite at 31.9 ± 0.5 to 32.7 ± 0.3 Ma. The temporal and spatial correlation of these magmatic phases with the geological evolution of the Biga Peninsula suggests that they have been emplaced during post-collisional tectonics and magmatism. In addition, trace element geochemistry indicates that the magmatic rocks of the Kirazlı deposit have been sourced from a subduction-modified metasomatized mantle, and have been affected by AFC processes and/or crustal contamination.

The porphyry Cu orebody has been emplaced during the late Eocene and has been overprinted by base metal and molybdenite events. Later, a younger HS epithermal system overprinted the porphyry Cu orebody and the base metal and molybdenite events at shallow depths. Detailed structural studies in the Kirazlı deposit are also recommended to clarify the exact tectonic evolution from porphyry Cu ore formation at ca. 36.7 Ma to HS epithermal emplacement at ca. 30.7 Ma.

Our radiometric ages at the Kirazlı deposit document that there is no magmatic and metallogenic gap between magmatic phases defined at 42–35 Ma and 30–18 Ma in previous studies for the Biga Peninsula. This indicates continuous hydrothermal and magmatic evolution in the Biga Peninsula throughout the late Eocene to Miocene, like in the adjacent Bulgarian and Greek Rhodope Massif.

## Supplementary informatio﻿n

ESM 1: Tables ESM 2: Figures ESM 3: Detailed Methodology ESM 4: References
