# Supplementary material for: Protracted metallogenic and magmatic evolution of the Kirazlı epithermal Au-Ag and porphyry Cu deposits, Biga Peninsula, NW Turkey: evidence from zircon U-Pb, muscovite 40Ar/39Ar, and molybdenite Re-Os geochronology
Source: Miner Depos. 2023 Dec 18;59(5):885–905. doi: 10.1007/s00126-023-01235-2 (PMC11102863; doi:10.1007/s00126-023-01235-2)
Supplement: Supplementary file 2 — ESM 2: Figures [file 126_2023_1235_MOESM2_ESM.docx]

# Protracted metallogenic and magmatic evolution of the epithermal Au-Ag and porphyry Cu deposits at the Kirazlı district, Biga Peninsula, NW Turkey: Evidence from zircon U-Pb, muscovite ^40^Ar/^39^Ar, and molybdenite Re-Os geochronology

Ali Aluç, İlkay Kuşcu, Alexey Ulyanov, David Selby, Clémentine Antoine, Richard Spikings, Robert Moritz

**Figure S1**. Photomicrographs of plagioclase-phyric andesite lava flow. (A) skeletal magmatic quartz with pseudo crystal of hornblende(?), (B) moderately altered zoned plagioclase, (C) muscovite replacing intensely altered plagioclase. Photomicrographs of basaltic andesite lava flow. (D) magmatic quartz with intensely altered plagioclase, (E) pseudo-crystal of the pyroxene, (F) muscovite form in the crystal cage of the altered hornblende. Abbreviations ms: muscovite, p-hb: pseudo-crystal of hornblende, biotite, qtz: quartz, plag: plagioclase, p-px: pseudo-crystal of pyroxene.

**Figure S2**. Graphical representation of obtained radiometric data from Kirazlı district. Alunite ^40^Ar/^39^Ar age from Yiğit (2012)


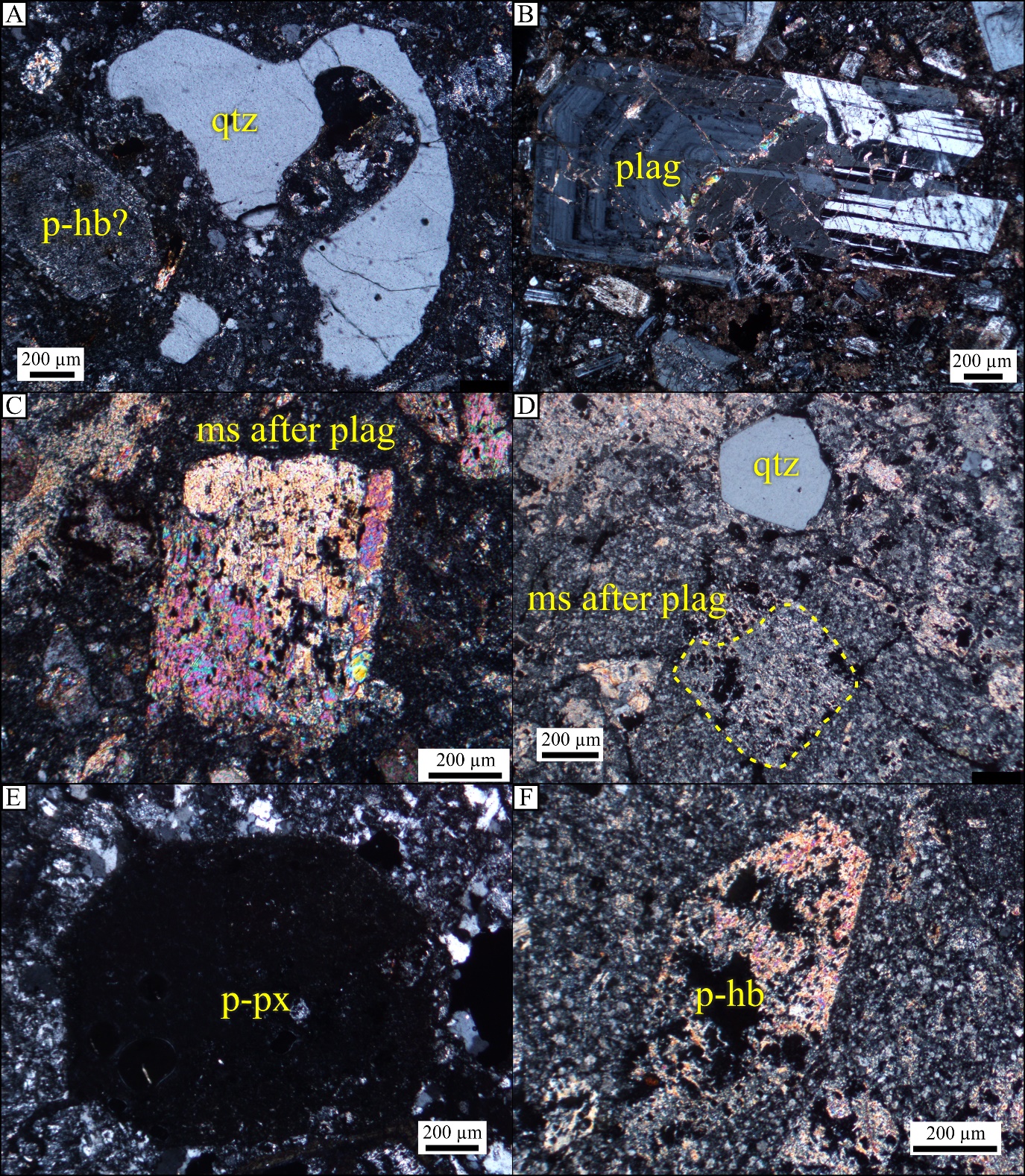


Figure S1. Photomicrographs of plagioclase-phyric andesite lava flow. (A) skeletal magmatic quartz with pseudo crystal of hornblende(?), (B) moderately altered zoned plagioclase, (C) muscovite replacing intensely altered plagioclase. Photomicrographs of basaltic andesite lava flow. (D) magmatic quartz with intensely altered plagioclase, (E) pseudo-crystal of the pyroxene, (F) muscovite form in the crystal cage of the altered hornblende. Abbreviations ms: muscovite, p-hb: pseudo-crystal of hornblende, biotite, qtz: quartz, plag: plagioclase, p-px: pseudo-crystal of pyroxene.


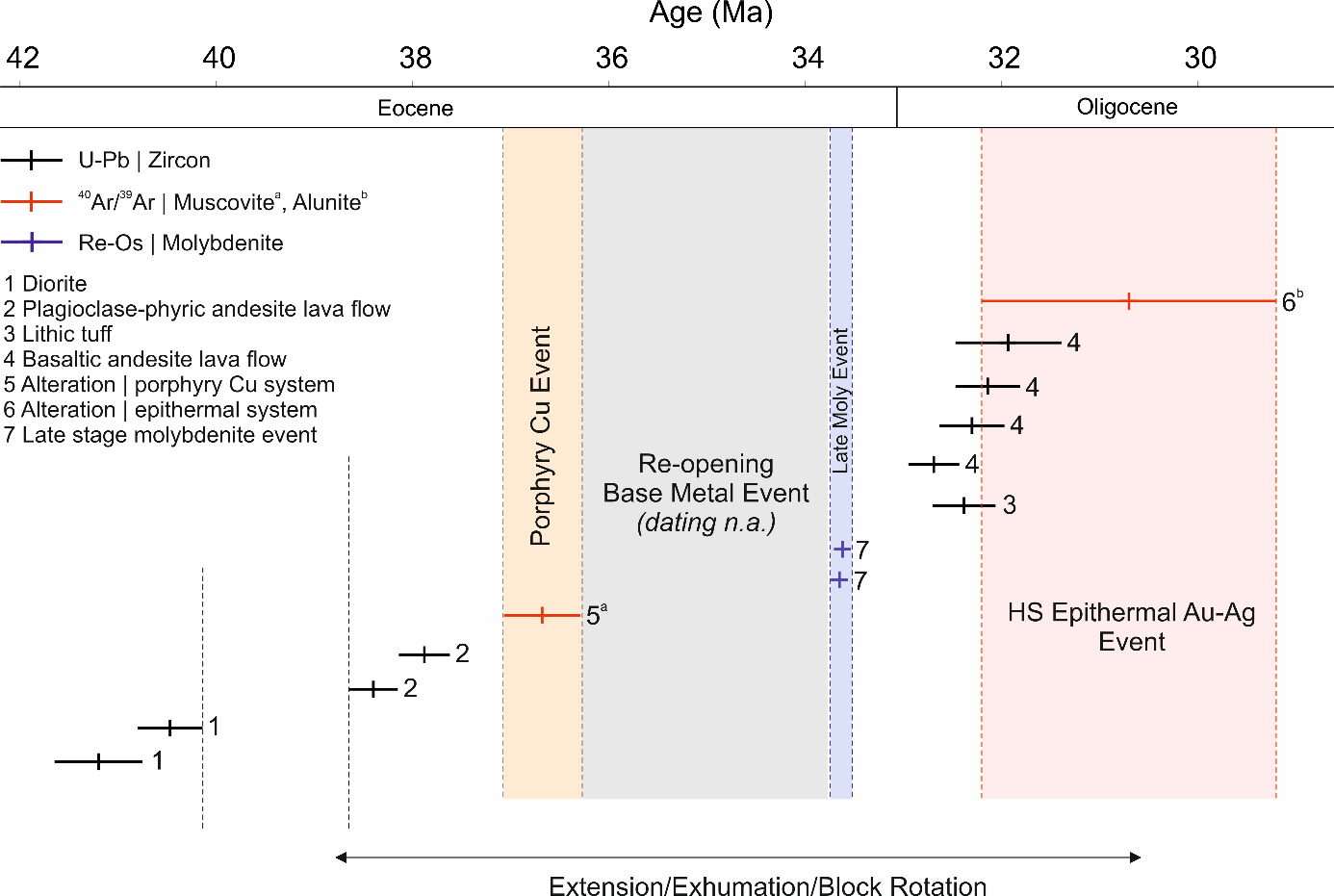


Figure S2. Graphical representation of obtained radiometric data from Kirazlı district. Alunite ^40^Ar/^39^Ar age from Yiğit (2012).
