# Supplementary material for: Protracted metallogenic and magmatic evolution of the Kirazlı epithermal Au-Ag and porphyry Cu deposits, Biga Peninsula, NW Turkey: evidence from zircon U-Pb, muscovite 40Ar/39Ar, and molybdenite Re-Os geochronology
Source: Miner Depos. 2023 Dec 18;59(5):885–905. doi: 10.1007/s00126-023-01235-2 (PMC11102863; doi:10.1007/s00126-023-01235-2)
Supplement: Supplementary file 3 — ESM 3: Detailed Methodology [file 126_2023_1235_MOESM3_ESM.docx]

# Protracted metallogenic and magmatic evolution of the epithermal Au-Ag and porphyry Cu deposits at the Kirazlı district, Biga Peninsula, NW Turkey: Evidence from zircon U-Pb, muscovite ^40^Ar/^39^Ar, and molybdenite Re-Os geochronology

Ali Aluç, İlkay Kuşcu, Alexey Ulyanov, David Selby, Clémentine Antoine, Richard Spikings, Robert Moritz

# Detailed Methodology

Fifteen rock samples from different plutonic and volcanic suites of the Kirazlı district were selected for whole-rock geochemistry. Samples were crushed and powdered using a hydraulic press and an agate mill. Lithium tetraborate fused pallets were prepared for each sample and major elements were analyzed by X-ray fluorescence (XRF) using a Philips PW 2400 and PANalytical AXIOS^mAX^ spectrometers at the University of Lausanne. Trace elements, including rare earth elements (REE), were determined using an Element XR sector-field ICP-MS (Thermo Scientific) interfaced to a RESOlution SE 193 nm excimer ablation system equipped with a 2-vol. S155 ablation cell (Australian Scientific Instruments) at the University of Lausanne. The operational parameters of the laser included a spot diameter of 100 μm, a repetition rate of 10 Hz, and an on-sample energy density of ⁓ 6 J/cm^2^. Helium was used as the ablation cell gas. The NIST SRM 612 glass was used for external standardization of the relative sensitivity factors and the XRF values of CaO - for internal standardization. Three ablation spots were collected on each sample and the data reduction processes were performed using LAMTRACE (Jackson 2008).

Traditional mineral separation techniques (crushing, milling, shaking table, magnetic separation, and heavy liquid) were applied to extract zircon grains from nine samples for U-Pb geochronology. Gem-quality zircon grains (n ≈ 30 for each sample) were handpicked, mounted in epoxy resin, and polished. Before LA-ICP-MS analyses, cathodoluminescence (CL) images were taken to define zoning and select the location of the laser spots, using a CamScan MV 2300 SEM operated at an acceleration voltage of 10 kV, a probe current of 0.5 nA, and a working distance of *40 mm at the University of Geneva. Age dating was performed using the same ICP spectrometer and ablation system for the analysis of lithium tetraborate pellets. Ablation settings included a repetition rate of 5 Hz and an on-sample energy density of 3 J/cm^2^; analytical conditions were similar to those described by Ulianov et al. (2012) at the University of Lausanne. The analytical spot diameter varied between 20 and 24 μm according to grain size and growth structure complexity. GJ-1 (Griffin et al. 2004; Jackson et al. 2004) and Plešovice (Sláma et al. 2008) were used as primary and secondary natural zircon standards, respectively, to assess the relative sensitivity and accuracy. Raw data were processed offline using the LAMTRACE software (Jackson 2008). The weighted mean age diagrams showing the ages calculated from U–Pb isotope ratios and U–Pb frequency plots were generated using the Isoplot/Ex v. 4.15 software (Ludwig 2012) after excluding discordant and outlier data.

Two molybdenite samples were extracted from quartz-pyrite-molybdenite veins (Figs. 6E and 6H) within sericite-rich alteration assemblages in the Kale zone using traditional mineral separation techniques at the Mugla Sitki Kocman University for Re-Os dating. An average of 30mg of pure molybdenite for each sample were handpicked under a binocular to remove remaining impurities and sent to the University of Durham (U.K.) for molybdenite Re-Os geochronology in the Source Rock and Sulfide Geochemistry and Geochronology Laboratory. Detailed sample preparation and analytical protocols are documented by Selby and Creaser (2001; 2004), Selby et al. (2007), and Lawley and Selby (2012). In brief, the ^187^Re and ^187^Os isotope ratios were determined using isotope-dilution Negative Thermal Ionization Mass Spectrometry on a Thermo Scientific TRITON mass spectrometer using static Faraday collection. The Carius tube method was used for the dissolution of molybdenite and equilibration of sample and tracer solution Re and Os using inverse aqua-regia. Osmium was isolated and purified from the aqua-regia using solvent extraction and micro distillation. Rhenium was purified by solvent extraction and anion chromatography. Total procedural blanks for Re and Os were 2 pg and 0.1 pg, respectively, with a ^187^Os/^188^Os blank composition of 0.17 ± 0.02 (n = 1). Rhenium and Osmium concentrations and Re-Os molybdenite date uncertainties are reported at the 2σ absolute level, which was determined through error propagation of uncertainties related to Re and Os mass spectrometer measurements, tracer calibration, sample and tracer solution weight, reproducibility of Re and Os standards, as well as uncertainties related to the blank determination. The Re-Os dates are calculated using ^187^Re decay constants from Smoliar et al. (1996).

For ^40^Ar/^39^Ar dating, the muscovite aliquot was weighed, wrapped in Cu foil and placed between Fish Canyon [Tuff](https://www.sciencedirect.com/topics/earth-and-planetary-sciences/tuff) (FCT) [sanidine](https://www.sciencedirect.com/topics/earth-and-planetary-sciences/sanidine) flux monitors in a linear stack in quartz tubes, and irradiated for 15 h in the Oregon State University TRIGA reactor using the shielded CLICIT site. The FCT-SAN monitors were positioned between every two aliquots in the linear stack, with a mean separation of ∼1 cm. After irradiation, the aliquot was heated to ∼100 °C for 24 h using a heat lamp. Thirty milligrams of aliquot were loaded into cleaned copper plates housed in a double-pumped viewport. Each sample was step-wise degassed with heating increments between 0.13 W and 0.40 W using a CO_2_-IR laser (10.4–10.8 μm) beam with a 2 mm diameter that was rastered over the entire sample to ensure even heating. Gas was cleaned using a hot GP50 (ST101) getter, cold NP10 (ST101), and cold finger held at −130 °C for ten minutes before expansion into the [mass spectrometer](https://www.sciencedirect.com/topics/earth-and-planetary-sciences/mass-spectrometer). Argon isotopes were measured on a multi-collector Thermo Scientific Argus VI mass spectrometer in static mode. Mass/charge 40, 39, 38, 37, and 36 were measured on Faraday collectors with 10^13^ Ω feedback resistors. The step-heating experiment included 14 heating steps, with a blank measurement after each analysis step. Automated peak centering was performed before every analytical (not including blank) step. Mass discrimination was estimated by running a sequence of air shots overnight every third day. Baseline and blank corrected data were further corrected for mass discrimination as functions of gas intensity from air shot measurements. All data regression was done using ArArCalc (Koppers, 2002) with reactor correction coefficients of (^39^Ar/^37^Ar)_Ca_ = (7.60 ± 0.09) × 10^−4^; (^36^Ar/^37^Ar)_Ca_ = (2.70 ± 0.02) × 10^−4^; and (^40^Ar/^39^Ar)_K_ = (7.30 ± 0.90) × 10^−4^ (Jourdan et al. 2007a, b, c), and with the ^40^K decay constant and branching coefficient of Steiger and Jäger (1977). J values were calculated by linear regression between each pair of measured monitors, using an FCT-SAN age of 28.201 ± 0.046 Ma (Kuiper et al. 2008), and a ^40^Ar/^36^Ar value of 298.56 ± 0.31 (Lee et al. 2006) assumed to be the composition of initial (atmospheric) Ar. All incremental data was blank-corrected.

# References

Griffin WL, Belousova EA, Shee SR, Pearson NJ, O’Reilly SY (2004) Archean crustal evolution in the northern Yilgarn Craton: U–Pb and Hf-isotope evidence from detrital zircons. Precambrian Res 131(3):231–282. <https://doi.org/10.1016/j.precamres.2003.12.011>

Jackson SE, Pearson NJ, Griffin WL, Belousova EA (2004) The application of laser ablation-inductively coupled plasma-mass spectrometry to in situ U–Pb zircon geochronology. Chem Geol 211(1):47–69. <https://doi.org/10.1016/j.chemgeo.2004.06.017>

Jackson SE (2008) LAMTRACE data reduction software for LA-ICP-MS. Laser Ablation ICP-MS in the Earth Sciences: Current Practices and Outstanding Issues, 40

Jourdan F, Feraud G, Bertrand H, Watkeys MK (2007a) From flood basalts to the inception of oceanization: example from the ^40^Ar/^39^Ar high-resolution picture of the Karoo large igneous province. Geochem Geophys Geosyst 8(2):n/a. https://doi.org/10.1029/2006GC001392

Jourdan F, Feraud G, Bertrand H, Watkeys MK, Renne PR (2007b) Distinct brief major events in the Karoo large igneous province clarified by new ^40^Ar/^39^Ar ages on the Lesotho basalts. Lithos 98(1-4):195–209. https://doi.org/10.1016/j.lithos.2007.03.002

Jourdan F, Matzel JP, Renne PR (2007c) ^39^Ar and ^37^Ar recoil loss during neutron irradiation of sanidine and plagioclase. Geochim Cosmochim Acta 71(11): 2791–2808. https://doi.org/10.1016/j.gca.2007.03.017

[Koppers AAP (2002](https://www.sciencedirect.com/science/article/pii/S0009254122003801#bb0100)) ArArCALC—software for ^40^Ar/^39^Ar age calculations. Computer Geosci 28(5):605–619. <https://doi.org/10.1016/S0098-3004(01)00095-4>

[Kuiper KF, Deino A, Hilgen FJ, Krijgsman W, Renne PR, Wijbrans JR (2008](https://www.sciencedirect.com/science/article/pii/S0009254122003801#bb0110)) Synchronizing Rock Clocks of Earth History. Sci 320(5875): 500–504. https://doi/10.1126/science.1154339

Lawley CJM, Selby D (2012) Re-Os geochronology of quartz-enclosed ultrafine molybdenite: implications for ore geochronology. Econ Geol 107(7): 1499–1505. <https://doi.org/10.2113/econgeo.107.7.1499>

Lee JY, Marti K, Severinghaus JP, Kawamura K, Yoo HS, Lee JB, Kim JS (2006) A redetermination of the isotopic abundances of atmospheric Ar. Geochimi Cosmochim Acta 70(17): 4507–4512. https://doi.org/10.1016/j.gca.2006.06.1563

Ludwig KR (2012) Isoplot 3.75: A geochronological toolkit for Microsoft Excel. Spec. Publ no. 5, Berkeley Geochronology Center, Berkeley, California, p 75

Selby D, Creaser RA (2001) Re-Os Geochronology and Systematics in Molybdenite from the Endako Porphyry Molybdenum Deposit, British Columbia, Canada. Econ Geol 96(1):197–204. https://doi.org/10.2113/gsecongeo.96.1.197

Selby D, Creaser RA (2004) Macroscale NTIMS and microscale LA-MC-ICP-MS Re-Os isotopic analysis of molybdenite: Testing spatial restrictions for reliable Re-Os age determinations, and implications for the decoupling of Re and Os within molybdenite. Geochimi Cosmochim Acta 68(19):3897–3908. <https://doi.org/10.1016/j.gca.2004.03.022>

Selby D, Creaser RA, Stein HJ, Markey RJ, Hannah JL (2007) Assessment of the 187Re decay constant by cross calibration of Re–Os molybdenite and U–Pb zircon chronometers in magmatic ore systems. Geochimi Cosmochim Acta 71(8):1999–2013. <https://doi.org/10.1016/j.gca.2007.01.008>

Slama J, Kosler J, Crowley JL, Gerdes A, Hanchar J, Horstwood M, Morris GA, Nasdala L, Norberg N, Schaltegger U, Tubrett MN, Whitehouse MJ (2008) Plešovice zircon – a new natural reference material for U-Pb and Hf isotopic microanalysis. Chem Geol 249:1–35. <https://doi.org/10.1016/j.chemgeo.2007.11.005>

Smoliar MI, Walker RJ, Morgan JW (1996) Re-Os Ages of Group IIA, IIIA, IVA, and IVB Iron Meteorites. Sci 271(5252):1099. <https://doi.org/10.1126/science.271.5252.1099>

Steiger RH, Jager E (1977) Subcommission on geochronology: Convention on the use of decay constants in geo- and cosmochronology. Earth Planet Sci Lett 36(3):359–362. https://doi.org/10.1016/0012-821X(77)90060-

Ulianov A, Müntener O, Schaltegger U, Bussy F (2012) The data treatment dependent variability of U–Pb zircon ages obtained using mono-collector, sector field, laser ablation ICPMS. J Anal At Spectrom 27(4):663–676. https://doi.org/10.1039/C2JA10358C
