# Supplementary material for: Protracted metallogenic and magmatic evolution of the Kirazlı epithermal Au-Ag and porphyry Cu deposits, Biga Peninsula, NW Turkey: evidence from zircon U-Pb, muscovite 40Ar/39Ar, and molybdenite Re-Os geochronology
Source: Miner Depos. 2023 Dec 18;59(5):885–905. doi: 10.1007/s00126-023-01235-2 (PMC11102863; doi:10.1007/s00126-023-01235-2)
Supplement: Supplementary file 4 — ESM 4: References [file 126_2023_1235_MOESM4_ESM.docx]

# Protracted metallogenic and magmatic evolution of the epithermal Au-Ag and porphyry Cu deposits at the Kirazlı district, Biga Peninsula, NW Turkey: Evidence from zircon U-Pb, muscovite ^40^Ar/^39^Ar, and molybdenite Re-Os geochronology

Ali Aluç, İlkay Kuşcu, Alexey Ulyanov, David Selby, Clémentine Antoine, Richard Spikings, Robert Moritz

# References of the ESM 1

Altunkaynak S, Sunal G, Aldanmaz E, Genc CS, Dilek Y, Furnes H, Foland KA, Yang J, Yıldız M (2012) Eocene Granitic Magmatism in NW Anatolia (Turkey) revisited: New implications from comparative zircon SHRIMP U–Pb and 40Ar–39Ar geochronology and isotope geochemistry on magma genesis and emplacement. Lithos 155:289–309. https://doi.org/10.1016/j.lithos.2012.09.008

Aluç A, Gürler Z, Kuşcu I, Aydogan S (2014) A New Low Sulfidation Epithermal Au -Ag Mineralization within Biga Peninsula: Karadere (Burhaniye, Balıkesir, Turkey). The 8th International Symposium on Eastern Mediterranean Geology, Muğla Sıtkı Koçman University, Turkey, 13-17 October 2014, abstract, pp 103

Agdemir N, Kirikoglu MS, Lehmann B, Tietze J (1994) Petrology and alteration geochemistry of the epithermal Balya Pb-Zn-Ag deposit, NW Turkey: a reconnaissance study. Mineralium Deposita 29: 366-371 https://doi.org/10.1007/BF00191043

Aysal N (2015) Mineral chemistry, crystallization conditions and geodynamic implications of the Oligo–Miocene granitoids in the Biga Peninsula, Northwest Turkey. J Asian Earth Sci 105: 68-84 https://doi.org/10.1016/j.jseaes.2015.03.026

Beccaletto L, Bonev N, Bosch D, Bruguier O (2007) Record of a Palaeogene syn-collisional extension in the north Aegean region: evidence from the Kemer micaschists (NW Turkey). Geological Mag 144: 393-400 https://doi.org/10.1017/S001675680700310X.

Bonev N, Marchev P, Singer B (2006) ^40^Ar/^39^Ar geochronology constraints on the Middle Tertiary basement extensional exhumation, and its relation to ore-forming and magmatic processes in the Eastern Rhodopes (Bulgaria). Geodinamica Acta 19:267–282. <https://doi.org/10.3166/ga.19.267-282>

Bozkaya G, Banks DA (2015) Physico-chemical controls on ore deposition in the Arapucandere Pb–Zn–Cu-precious metal deposit, Biga Peninsula, NW Turkey. Ore Geol Rev 66:65–81. https://doi.org/10.1016/j.oregeorev.2014.10.014

Brunetti P (2016) Magmatic-hydrothermal evolution and post-ore modifications of the Halilağa porphyry Cu-Au deposit, NW Turkey. Dissertation, University of British Columbia

Cicek M, Oyman T (2016) Origin and evolution of hydrothermal fluids in epithermal Pb-Zn-Cu±Au±Ag deposits at Koru and Tesbihdere mining districts, Çanakkale, Biga Peninsula, NW Turkey. Ore Geol Rev 78:176–195. https://doi.org/10.1016/j.oregeorev.2016.03.020

Colakoglu AR (2000) Küçükdere (Havran-Balıkesir) epitermal altın damarının özellikleri. Geol Bull Turk 43(2): 99–110

Cormier A, Jutras M, Welhener H, Minard T, Chiaramello P, Cremeens J (2017a) NI 43-101 Technical Report Feasibility Study Technical Report on the Kirazlı Project, Çanakkale Province, Turkey, p 420

Cormier A, Jutras M, Welhener H, Minard T, Chiaramello P, Cremeens J (2017b) NI 43-101 Technical Report Feasibility Study Technical Report on the Ağıdağı and Çamyurt Project, Çanakkale Province, Turkey, p 551

Delaloye M, Bingöl E (2000) Granitoids from Western and Northwestern Anatolia: Geochemistry and Modeling of Geodynamic Evolution. Int Geol Rev 42: 241-268 https://doi.org/10.1080/00206810009465081.

Eleftheriadis G, Lippolt HJ (1984) Altersbestimmungen zumoligozanen Vulkanismus der Sud-Rhodopen/Nord Griechen land. Neues Jahrbuch für Geologie und Palaontologie, Monatshefte 3:179 – 191

Erkul F, Sozbilir H, Helvaci C (2005) Stratigraphy and Geochronology of the Early Miocene Volcanic Units in the Bigadiç Borate Basin, Western Turkey . Turk J Earth Sci 14(3):227–253

Georgiev V, Milovanov P, Monchev P (2003) K–Ar dating of the magmatic activity in the Momchilgrad volcano-tectonic depression. Comptes Rendus de l‘Acade´mie Bulgare des Sciences 56:49–54

Golder Associates (2017a) Supplementary Information Package - Ivrindi Gold and Silver Mine Project, Golder Associates, Ankara, Turkey, p 221

Golder Associates (2017b) Supplementary Information Package - Lapseki Gold and Silver Mine Project, Golder Associates, Ankara, Turkey, p 239

Grieve PL (2007) NI 43-101 Technical report on the Pirentepe and Halilağa Properties, Çanakkale, Western Anatolia, Turkey, p 172

Gulyuz N, Shipton ZK, Kuşcu I, Lord RA, Kaymakci N, Gulyuz E, Gladwell DR (2018) Repeated reactivation of clogged permeable pathways in epithermal gold deposits: Kestanelik epithermal vein system, NW Turkey. J Geol Soc 175:509–524. https://doi.org/10.1144/jgs2017-039

Harkovska A, Marchev P, Machev Ph, Pecskay Z (1998) Paleogene magmatism in the Central Rhodope area, Bulgaria — a review and new data. Acta Vulcanologica 10:199–216

Innocenti F, Kolios N, Manetti P, Mazzuoli R, Peccerillo A, Rita F, Villari L (1984) Evolution and geodynamic significance of the Tertiary orogenic volcanism in northeastern Greece. Bull Volcanologique 47:25–37

Kaiser-Rohrmeier M, Handler R, Von Quadt A, Heinrich C (2004) Hydrothermal Pb–Zn ore formation in the central Rhodopian dome, south Bulgaria: review and new time constraints from Ar–Ar geochronology. Swiss Bull Miner Petr 84:37–58.

Kaiser-Rohrmeier M, Von Quadt A, Driesner T, Heinrich CA, Handler R, Ovtcharova M, Ivanov Z, Petrov P, Sarov St, Peytcheva I (2013) Post-Orogenic Extension and Hydrothermal Ore Formation: High-Precision Geochronology of the Central Rhodopian Metamorphic Core Complex (Bulgaria-Greece). Econ Geol 108(4):691–718. <https://doi.org/10.2113/econgeo.108.4.691>

Kuşcu I, Tosdal RM, Kuşcu G (2019a) Episodic porphyry Cu (-Mo-Au) formation and associated magmatic evolution in Turkish Tethyan collage. Ore Geol Rev 107:119–154. https://doi.org/10.1016/j.oregeorev.2019.02.005

Kuşcu I, Tosdal RM, Kuşcu G (2019b) Porphyry-Cu Deposits of Turkey. In: Pirajno F, Unlu T, Donmez C, Sahin MB (eds) Mineral Resources of Turkey, 1^st^ edn. Springer Nature, Switzerland, pp 337–425. https://doi.org/10.1007/978-3-030-02950-0

Leroux GM (2016) Stratigraphic and petrographic characterization of HS epithermal Au-Ag mineralization at the TV Tower district, Biga Peninsula, NW Turkey. Dissertation, University of British Columbia

Lilov P, Yanev Y, Marchev P (1987) K/Ar dating of the Eastern Rhodopes Paleogene magmatism. Geologica Balcanica 17:49–58

Magganas A, Bigazzi G, Kyriakopoulos K, Balestrieri ML (2004) Low-T thermochronological evolution of the Vrondou composite pluton (northern Greece) using apatite fission track analyses. In: Chatzipetros A, Pavlides S (Eds.) Proceedings of 5th International Symposium on Eastern Mediterranean Geology, Thessaloniki, Greece 3:1165–1168

Marakis G (1969) Geochronology studies of some granites from Macedonia. Annales Geologiques des Pays Helleniqes 21:121–152

Marchev P, Kaiser-Rohrmeier M, Heinrich C, Ovtcharova M, Von Quadt A, Raicheva R (2005) Hydrothermal ore deposits related to post-orogenic extentional magmatism and core complex formation: the Rhodope Massif of Bulgaria and Greece. Ore Geol Rev 27:53–89. <https://doi.org/10.1016/j.oregeorev.2005.07.027>

Marchev P, Kibarov P, Spikings R, Ovtcharova M, Marton I, Moritz R (2010) ^40^Ar/^39^Ar and U-Pb geochronology of the IranTepe volcanic complex, Eastern Rhodopes. Geologica Balcanica 39:3–12. <https://doi.org/10.52321/GeolBalc.39.3.3>

Marchev P, Singer B (2002) ^40^Ar/^39^Ar geochronology of magmatism and hydrothermal activity of the Madjarovo base-precious metal ore district, eastern Rhodopes, Bulgaria. In: Blundell DJ, Neubauer F, Von Quadt A (eds) The Timing and Location of Major Ore Deposits in An Evolving Orogen. Geol Soc London Spec Pub 204:137–150. <https://doi.org/10.1144/GSL.SP.2002.204.01.09>

Marton I, Moritz R, Spikings R (2010) Application of low-temperature thermochronology to hydrothermal ore deposits: formation, preservation and exhumation of epithermal gold systems from the Eastern Rhodopes, Bulgaria. Tectonophysics 483:240–254. <https://doi.org/10.1016/j.tecto.2009.10.020>

Moritz R, Noverraz C, Marton I, Marchev P, Spikings R, Fontignie D, Spangenberg JE, Vennemann T, Kolev K, Hasson S (2014) Sedimentary-rock-hosted epithermal systems of the Tertiary Eastern Rhodopes, Bulgaria: new constraints from the Stremtsi gold prospect. Geol Soc London Spec Pub 402:207–230. <https://doi.org/10.1144/SP402.7>

Murakami H, Watanabe Y, Stein H (2005) Re-Os ages for molybdenite from the Tepeoba breccia-centered Cu-Mo-Au deposit, western Turkey: Brecciation-triggered mineralization. Mineral Deposit Research: Meeting the Global Challenge, Berlin, Heidelberg

Ortelli M, Moritz R, Voudouris P, Cosca M, Spangenberg J (2010) Tertiary porphyry and epithermal association of the Sapes-Kassiteres district, Eastern Rhodopes, Greece. In: Goldfarb R, Marsh E, Monecke T (eds) The Challenge of Finding New Mineral Resources. SEG Conference, Keystone, Colorado, U.S.A.

Ovtcharova M, Von Quadt A, Heinrich CA, Frank M, Kaiser-Rohmeier M, Peytcheva I, Cherneva Z (2003) Triggering of hydrothermal ore mineralization in the Central Rhodopean Core Complex (Bulgaria) - Insight from isotope and geochronological studies on Tertiary magmatism and migmatization. In: Eliopoulos DG, Allan C (eds) Mineral Exploration and Sustainable Development, Proceedings of the 7th Biennial SGA Meeting. Athens, Greece, pp 367–370.

Oyman T (2010) Geochemistry, mineralogy and genesis of the Ayazmant Fe–Cu skarn deposit in Ayvalik, (Balikesir), Turkey. Ore Geol Rev 37: 175-201 https://doi.org/10.1016/j.oregeorev.2010.03.002.

Özpinar Y, Kiliç M, Sari R, Agnerian H, Doygun Z (2012) A new assessment of the Kisacik gold mineralization (Ayvacik-Çanakkale; NW Anatolia). Roman J Earth Sci 86(2): 111‐116.

Pecskay Z, Eleftheriadis G, Koroneos A, Soldatos T, Christofides G (2003) K/Ar dating, geochemistry and evolution of the Tertiary volcanic rocks (Thrace, northeastern Greece). In: Eliopoulos D (Eds.) Mineral Exploration and Sustainable Development. Millpress, Rotterdam, pp 1229 – 1232

Pecskay Z, Harkovska A, Hadjiev A (2000) K–Ar dating of Mesta volcanics (SW Bulgaria). Geologica Balcanica 30:3–11.

Sánchez MG, McClay KR, King AR, Wijbrams JR (2016) Cenozoic Crustal Extension and Its Relationship to Porphyry Cu-Au-(Mo) and Epithermal Au-(Ag) Mineralization in the Biga Peninsula, Northwestern Turkey. In: Richards JP (ed) Tectonics and Metallogeny of the Tethyan Orogenic Belt Vol 19. Society of Economic Geologists. https://doi.org/10.5382/SP.19.05

Singer B, Marchev P (2000) Temporal evolution of arc magmatism and hydrothermal activity, including epithermal gold veins, Borovitsa caldera, southern Bulgaria. Econ Geol 95:1155–1164. <https://doi.org/10.2113/gsecongeo.95.5.1155>

Smith MT, Lepore WA, Incekaraoglu T, Boran H, Barrios A, Leroux GM, Ross K, Büyüksolak A, Sevimli A, Raabe K (2016) High-Sulfidation Epithermal Au and Porphyry Cu-Au Mineralization at the Karaayi Target, Biga Peninsula, Northwestern Turkey. In: Richards JP (ed) Tectonics and Metallogeny of the Tethyan Orogenic Belt Vol 19. Society of Economic Geologists. https://doi.org/10.5382/SP.19.04

Smith MT, Lepore WA, Incekaraoğlu T, Shabestari P, Boran H, Raabe K (2014) Küçükdağ: A New, High Sulfidation Epithermal Au-Ag-Cu Deposit at the TV Tower Property in Western Turkey. Econ Geol 109(6):1501–1511. https://doi.org/10.2113/econgeo.109.6.1501

Tunc IO, Yiğitbaş E, Şengün F, Wazeck J, Hofmann M, Linnemann U (2012) U-Pb zircon geochronology of northern metamorphic massifs in the Biga Peninsula (NW Anatolia-Turkey): new data and a new approach to understand the tectonostratigraphy of the region. Geodin Acta 25:202–225. https://doi.org/10.1080/09853111.2013.877242

Unal-Imer E, Gülec N, Kuşcu I, Fallick AE (2013) Genetic investigation and comparison of Kartaldag and Madendag epithermal gold deposits in Canakkale, NW Turkey. Ore Geol Rev 53:204–222. https://doi.org/10.1016/j.oregeorev.2013.01.009

Voudouris PC, Melfos V, Spry PG, Moritz R, Papavassiliou C, Falalakis G (2011) Mineralogy and geochemical environment of formation of the Perama Hill high-sulﬁdation epithermal Au–Ag–Te–Se deposit, Petrota Graben, NE Greece. Miner Petr 103:79–100. <https://doi.org/10.1007/s00710-011-0160-z>

Yanev Y, Stoykov S, Pecskay Z (1998) Petrology and K–Ar dating of the Paleogene magmatism in the region of the villages Yabalkovo and Stalevo, Eastern Rhodopes volcanic area. Bulgarian Academy of Sciences, Bull Geol Inst Series Geochem Miner Petr 34:97–110

Yiğit O (2012) A prospective sector in the Tethyan Metallogenic Belt: Geology and geochronology of mineral deposits in the Biga Peninsula, NW Turkey. Ore Geol Rev 46:118–148. https://doi.org/10.1016/j.oregeorev.2011.09.015

Yilmaz H (2003) Exploration at the Kuscayiri Au (Cu) prospect and its implications for porphyry-related mineralization in western Turkey. J Geochem Explor 77:133–150. https://doi.org/10.1016/S0375-6742(02)00274-1

Yilmaz H, Oyman T, Sonmez FN, Arehart GB, Billor Z (2010) Intermediate sulfidation epithermal gold-base metal deposits in Tertiary subaerial volcanic rocks, Sahinli/Tespih Dere (Lapseki/Western Turkey). Ore Geol Rev 37(3):236–258. <https://doi.org/10.1016/j.oregeorev.2010.04.001>

Yilmaz H, Sönmez F, Akay E, Sener A, Tufan S, (2013) Low-sulfidation epithermal Au-Ag mineralization in the Sindirgi District, Balikesir Province, Turkey. Turk J Earth Sci 22:485–522. https://doi.org/10.3906/yer-1204-10

Zagortchev I, Moorbath S, Lilov P (1987) Radiogeochronologic data on the Alpine magmatism in the western part of the Rhodope Massif. Geologica Balcanica 17:59–71
